# Mechanism of Reduction of Aqueous U(V)-dpaea and Solid-Phase U(VI)-dpaea Complexes: The Role of Multiheme *c*-Type Cytochromes

**DOI:** 10.1021/acs.est.3c00666

**Published:** 2023-05-03

**Authors:** Margaux Molinas, Karin Lederballe Meibom, Radmila Faizova, Marinella Mazzanti, Rizlan Bernier-Latmani

**Affiliations:** † Environmental Microbiology Laboratory, Ecole Polytechnique Fédérale de Lausanne (EPFL), Lausanne 1015, Switzerland; ‡ Group of Coordination Chemistry, Ecole Polytechnique Fédérale de Lausanne (EPFL), Lausanne 1015, Switzerland

**Keywords:** pentavalent U, solid phase U(VI), reduction mechanism, bioremediation, extracellular electron transfer

## Abstract

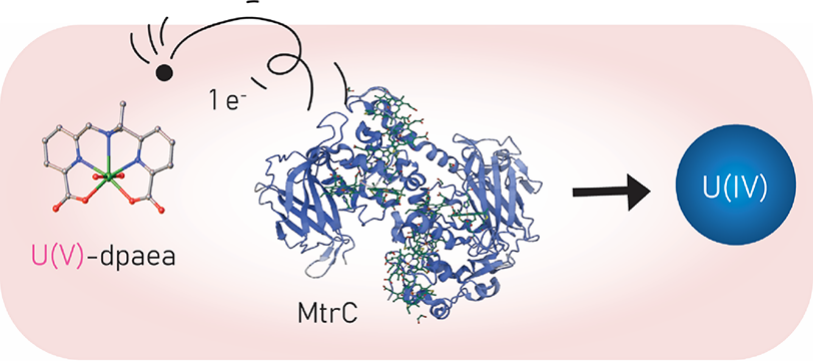

The biological reduction of soluble U(VI) complexes to form immobile U(IV) species has been proposed to remediate contaminated sites. It is well established that multiheme *c*-type cytochromes (MHCs) are key mediators of electron transfer to aqueous phase U(VI) complexes for bacteria such as *Shewanella oneidensis* MR-1. Recent studies have confirmed that the reduction proceeds via a first electron transfer forming pentavalent U(V) species that readily disproportionate. However, in the presence of the stabilizing aminocarboxylate ligand, dpaea^2–^ (dpaeaH_2_=bis(pyridyl-6-methyl-2-carboxylate)-ethylamine), biologically produced U(V) persisted in aqueous solution at pH 7. We aim to pinpoint the role of MHC in the reduction of U(V)-dpaea and to establish the mechanism of solid-phase U(VI)-dpaea reduction. To that end, we investigated U-dpaea reduction by two deletion mutants of *S. oneidensis* MR-1–one lacking outer membrane MHCs and the other lacking all outer membrane MHCs and a transmembrane MHC–and by the purified outer membrane MHC, MtrC. Our results suggest that solid-phase U(VI)-dpaea is reduced primarily by outer membrane MHCs. Additionally, MtrC can directly transfer electrons to U(V)-dpaea to form U(IV) species but is not strictly necessary, underscoring the primary involvement of outer membrane MHCs in the reduction of this pentavalent U species but not excluding that of periplasmic MHCs.

## Abstract 2

Microbial uranium reduction is a strategy for bioremediation of the subsurface. Here, we show the enzymatic mechanism of reduction of an intermediate valence state, pentavalent uranium.

## Introduction

Uranium (U) contamination in the subsurface, resulting from past or present anthropogenic activities such as mining, ore processing, and the production of weapons-grade, can be remediated biologically.^1−3^ To that end, subsurface microorganisms are stimulated to transform the highly mobile hexavalent U (U(VI)) species to less mobile tetravalent U (U(IV)) species.^4−8^ U(VI) and U(IV) are the most abundant oxidation states of U in the subsurface.

The biological reduction of metals and metalloids by *Shewanella oneidensis* MR-1 is an anaerobic process by which electrons, released upon oxidation of the electron donor, are transported along an electron transport chain, consisting of a sequence of *c*-type cytochromes^9^ linking the cytoplasm to the extracellular environment. Thus, electrons from the cytoplasm are delivered to a periplasmic pool of *c*-type cytochromes that shuttle them across the periplasm to, for instance, the *c*-type cytochrome chain MtrA-MtrC. The latter is part of the MtrCAB porin complex embedded in the outer membrane. The decaheme *c*-type cytochromes MtrC and OmcA, located on the cell outer membrane and on its finger-like extensions,^10^ are terminal reductases that deliver electrons to extracellular electron acceptors^9,11−13^ or to secreted flavin electron shuttles.^14−17^

The role of MHCs in the reduction of aqueous U(VI) was studied both experimentally by Marshall et al.^18^ and theoretically by Sundararajan et al.^19^ Marshall et al. investigated a collection of *S. oneidensis* MR-1 mutants to determine which *c*-type cytochromes were involved in the reduction of aqueous U(VI)-carbonate species. The mutant lacking all *c*-type cytochromes (Δ*ccmC*) lost the ability to reduce U(VI), underscoring the key role of these proteins in U(VI) reduction. Moreover, single outer membrane MHC deletion mutants Δ*mtrC* and Δ*omcA* and also the double cytochrome deletion mutant Δ*mtrC-omcA* displayed slower reduction rates as compared to wild-type (WT) MR-1. Such results suggest that both MtrC and OmcA are involved in electron transfer to aqueous-phase U(VI)-carbonate. Additionally, using purified MtrC and OmcA, they observed that MtrC, but not OmcA, could transfer electrons to U(VI)-citrate. However, the fact that single and double outer membrane MHC deletion mutants could still reduce U(VI)-carbonate hinted at periplasmic MHC-mediated or MtrF-mediated electron transfer. Furthermore, Sundararajan et al. studied the mechanism of aqueous uranyl(VI) reduction by the *c*-type cytochrome PpcA from *Geobacter sulfurreducens* using density functional theory (DFT) calculations.^19^ According to their model, a single electron transfer is expected to occur from PpcA to U(VI), producing U(V), followed by the spontaneous disproportionation of two U(V) to U(VI) and U(IV).

Recent studies have revealed the persistence of an aqueous pentavalent U (U(V))–organic ligand complex at circumneutral pH values under laboratory conditions.^20,21^ The ligand in question, dpaea^2–^ (dpaeaH_2_=bis(pyridyl-6-methyl-2-carboxylate)-ethylamine), is synthetic but belongs to the family of aminocarboxylate ligands. These ligands occur in the environment either naturally, for instance, dipicolinic acid (dpa) in bacterial endospores,^22,23^ or as a consequence of human-related activities such as metal chelation in remediation processes,^24^ radionuclide extraction in nuclear wastes,^25,26^ or additives in detergents.^27,28^ We have recently demonstrated that reduction of U(V)-dpaea can occur via a biological one electron transfer and not via disproportionation, as observed in systems containing carbonate-complexed uranyl.^29,30^ This suggests that upon stabilization of U(V), its biological reduction was possible. In fact, the reduction of solid-phase U(VI)-dpaea occurs via two single consecutive electron transfers: (i) solid phase U(VI)-dpaea is reduced to soluble U(V)-dpaea, and (ii) U(V)-dpaea to solid-phase U(IV) species, such as U(IV)-dpaea_2_, and non-crystalline U(IV).^21^ The site of reduction of U(V), whether at the outer membrane or in the periplasm, and its mechanism remain unknown.

While the reduction of aqueous U(VI) species has been abundantly documented, there is limited information about solid-phase U(VI) reduction. By analogy to solid-phase iron reduction,^11−13,31^ outer membrane MHCs are expected to be involved in this process in *S. oneidensis* MR-1. However, because solid-phase U(VI) is typically more soluble than ferric iron oxides, both direct reduction of the U(VI) solid phase by outer membrane MHCs^32^^,^^33^ and reduction after dissolution^27^ have been reported. In addition, ferric iron reduction can potentially occur via soluble electron shuttles such as flavins.^14,17,35,36^ However, flavins were previously shown not to be involved in U(VI) reduction.^16^ Furthermore, a kinetic model of the dissolution of sodium boltwoodite crystals (NaUO_2_SiO_3_OH·1.5 H_2_O) embedded within alginate beads was developed to describe how U(VI) crystals trapped into sediments micropores (inaccessible to bacteria) could be biologically reduced by *S. oneidensis* MR-1 (such as at the Hanford site, USA).^34^ The researchers concluded that the dissolution of U(VI) crystals and U(VI) diffusion out of the alginate beads was the *sine qua non* condition for it to become bioavailable for reduction by *S. oneidensis* MR-1, precluding a role for soluble electron shuttles in this process.

Additionally, while the role of MHCs in U(V) reduction has been exemplified in our previous work by showing the absence of reduction of U(V) by a Δ*ccmG* mutant (lacking all MHCs), it is unclear which MHCs are implicated, whether outer membrane, periplasmic, and/or cytoplasmic.

In this context, we sought to probe the involvement of outer membrane MHCs in solid-phase U(VI)-dpaea reduction to aqueous U(V)-dpaea as well as in the subsequent electron transfer to aqueous U(V)-dpaea. We investigated the mechanism of reduction of U(VI)-dpaea and aqueous U(V)-dpaea at the protein level by constructing two deletion mutants: one lacking the outer membrane MHCs MtrC, OmcA, and MtrF (strain Δ*mtrC/omcA/mtrF*, abbreviated ΔOMC) and one lacking the three outer membrane MHCs as well as MtrA (strain Δ*mtrC/omcA/mtrF/mtrA*, abbreviated ΔOMCΔMtrA)*.* These engineered strains were characterized using aqueous Fe(III)-citrate and solid ferrihydrite, and the observed results helped guide interpretation of U(VI)-dpaea and aqueous U(V)-dpaea reduction. Hence, using these mutants, we established that solid-phase U(VI)-dpaea reduction to U(V)-dpaea proceeds via outer membrane MHCs, through dissolution of the solid U(VI)-dpaea to aqueous U(VI)-dpaea and subsequent reduction of the dissolved aqueous U(VI)-dpaea. In addition, we provide evidence that the reduction of U(V)-dpaea is mediated by both periplasmic and outer membrane MHCs.

## Experimental Methods

### U(V)-dpaea Reduction

As U(V) is highly sensitive to oxidation, all materials were left at least 24 h under vacuum prior to entering the anoxic chamber and allowed to equilibrate for 2–3 days under anoxic conditions before use. *S. oneidensis* MR-1 WT, ΔOMC, and ΔOMCΔMtrA (mutant constructs are described in Texts S1 and S2) were incubated with synthetic U(V)-dpaea, prepared as previously described (([K(2.2.2.cryptand)][UO_2_(dpaea)] *M*_W_ = 998.93 g mol^–1^),^20^ in anoxic modified WLP medium with 20 mM lactate. Strain growth and preparation for experiments are described in Text S3. The initial concentration of U was about 310 μM. All experiments were conducted in quadruplicate and a no-cell control experiment was performed in parallel. The no-cell control experiment consisted of U(V)-dpaea in anoxic modified WLP medium (identical to the one used for the cells) in the presence of 20 mM lactate. At specific times, aliquots of the incubations were filtered through a 0.2 μm PTFE filter (Whatman, Maidstone, United Kingdom). U was quantified by ICP-MS (Text S4). Cell viability of the quadruplicates was evaluated by streaking an aliquot of culture on LB agar plates.

### Reduction of U(VI)-dpaea

*S. oneidensis* MR-1 wild type (WT), ΔOMC, and ΔOMCΔMtrA were incubated, under non-growth conditions, in anoxic modified Widdel low phosphate medium (WLP) in the presence of solid phase U(VI)-dpaea ([UO_2_(dpaea)] *M*_W_ =583 g mol^–1^) at an equivalent aqueous concentration of 657 μM, synthesized as previously described,^20^ along with 20 mM lactate, the electron donor. ΔOMC and ΔOMCΔMtrA mutant strains were engineered as described in Texts S1 and S2. Prior to incubation, *S. oneidensis* MR-1 WT, ΔOMC, and ΔOMCΔMtrA cells were prepared as described in Text S3. The starting OD_600_ of the incubations was measured to be 1. The incubations were maintained in the dark at room temperature, inside the anaerobic chamber. At several time points, aliquots of the incubations were filtered through 0.2 μm PTFE filters (Whatman, Maidstone, United Kingdom). Similarly, the three abovementioned strains were incubated with aqueous phase U(VI)-dpaea obtained by filtering suspensions of solid-phase U(VI)-dpaea (0.2 μm) to remove the solid phase in modified WLP medium supplemented with 20 mM lactate. Cell viability was evaluated by streaking an aliquot of culture on Luria-Bertani (LB) agar plates. Each experimental condition was run in duplicate. U was quantified by inductively coupled plasma mass spectrometry (ICP-MS) (Text S4).

### Reaction of MtrC with U(V)-dpaea or U(IV)-citrate

In the glovebox, five reactions were initiated: (i) U(V)-dpaea in buffer A (Supplementary Information); (ii) U(V)-dpaea in buffer A with oxidized MtrC; (iii) U(V)-dpaea in buffer A with reduced MtrC; (iv) U(IV)-citrate in buffer A; (v) U(IV)-citrate in buffer A with oxidized MtrC. Reactions (i) and (iv) served to control the initial oxidation state of U, and in the case of U(V)-dpaea, to assess its stability over the experimental time. U(V)-dpaea powder was resuspended in buffer A to a final concentration of 300 μM. Aqueous U(IV)-citrate (200 μM) was obtained from the reduction of U(VI)-citrate by *S. oneidensis* MR-1. Both oxidized and reduced MtrC was prepared to a concentration of 300 μM. The reactions were initiated by mixing equal volumes of U and MtrC in buffer A ((ii), (iii), and (v))). Timepoints were collected by removing an aliquot from the reaction mixture and immediately loading it onto an ion-exchange chromatography (IEC) resin to separate U(VI) from U(IV).^21^ The heme redox status was probed before and after the reaction by UV–vis spectrophotometry to evaluate how they were influenced by the U species. The U concentration in the U(IV) and U(VI) fractions obtained by IEC was quantified by ICP-MS.

## Results

### Reduction of Ferrihydrite and Fe(III)-citrate by the Mutant Strains

In order to characterize the reduction capacity of the ΔOMC and ΔOMCΔMtrA strains, they were incubated with two Fe(III) substrates: (i) ferrihydrite (solid phase Fe(III)) or (ii) Fe(III)-citrate (aqueous Fe(III) complex). The experimental procedures for ferrihydrite synthesis and cell incubations with both Fe substrates are described in Texts S5 and S6, respectively. In both experiments, a high Fe(II) starting concentration was measured, an observation we attribute to Fe(III) photoreduction^37^ in the HCl-digested samples when stored unprotected from light. In the presence of Fe(III)-citrate, we observed rapid Fe(III) reduction in the incubations with the WT strain, with the reaction complete within 2 h (Figure S1A). In contrast, in the incubations with ΔOMC, the Fe(II) concentration rose slowly but steadily to 1379 μM after 48 h (Figure S1A). As for ΔOMCΔMtrA, it was fully impaired in its capacity to reduce Fe(III)-citrate for the first 24 h, but a slight increase in the Fe(II) concentration was observed subsequently, up to 647.5 μM after 48 h (Figure S1A). Because no measurable Fe(II) was detected in the first 24 h, we suggest that cell death and lysis likely account for the reduction of Fe(III)-citrate by OMCΔMtrA between 24 and 48 h. We observed that strain ΔOMCΔMtrA cell concentration decreased from about 10^8^ to 10^7^ cells/mL within 48 h (Figure S2A), which could be attributed to the toxicity of Fe(III)-citrate substrate to the cells, as was also evident for the WT and ΔOMC strains (Figure S2A).

For ferrihydrite, the Fe(II) concentration rose slightly over the experimental time in the no cell control, which is likely due to microbial activity in the starting material, which cannot be sterilized. As expected, no significant ferrihydrite reduction was observed with either ΔOMC or ΔOMCΔMtrA within 48 h, even though cell viability was still at about 10^7^ cells/mL (Figure S2B), whereas in the WT incubations, the Fe(II) concentration reached 1539 μM (Figure S1B). In summary, both ΔOMC and ΔOMCΔMtrA showed impairment in the reduction of Fe(III).

### Reduction of Solid-Phase U(VI)-dpaea by the Mutant Strains

**Figure 1:**
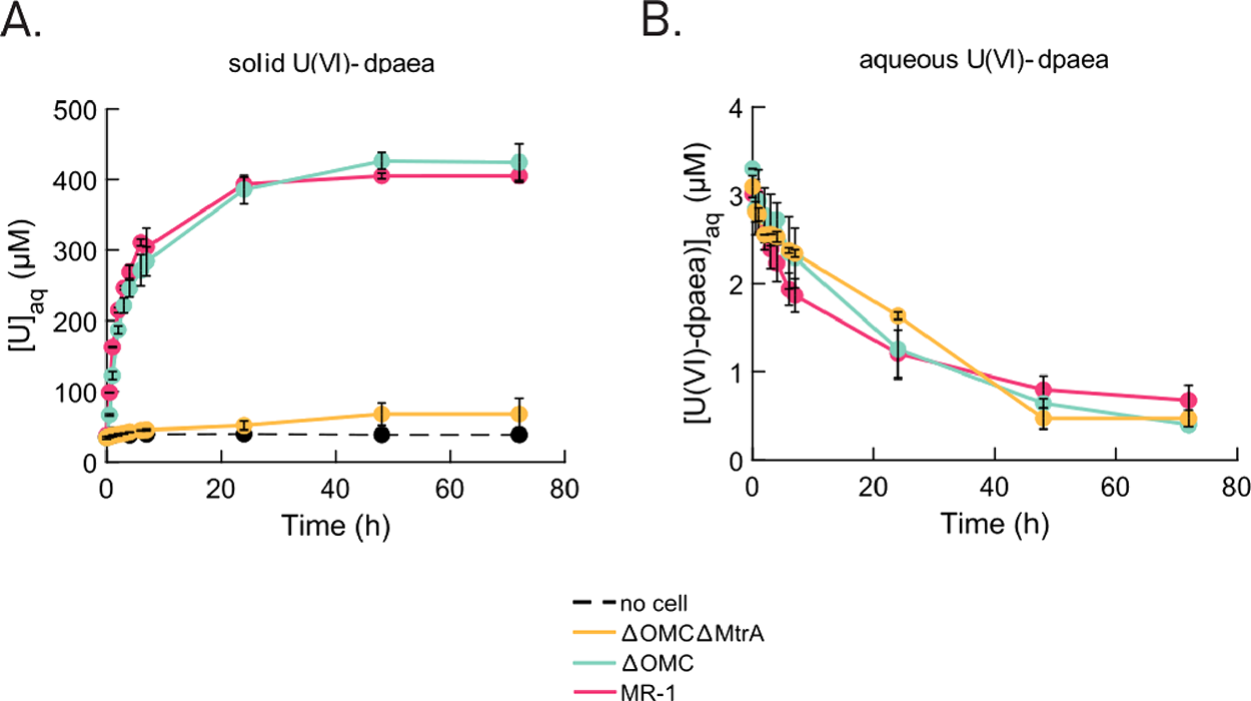
Strains MR-1 (pink), ΔOMC (blue), and ΔOMCΔMtrA (yellow) incubated with (A) U(VI) as ∼657 μM solid-phase U(VI)-dpaea or (B) U(VI) as 3 μM aqueous U(VI)-dpaea. Aqueous U concentration in the filtrate (measured by ICP-MS) is shown here up to 72 h evidencing (A) the increase in U(V) from the reduction of solid-phase U(VI)-dpaea to soluble U(V)-dpaea and (B) the decrease in aqueous U(VI) due to the formation of insoluble U(IV). The incubations were initially supplemented with 20 mM lactate as the electron donor, the cell density was OD_600_ = 0.1, and the pH value was 7.3. The error bars correspond to the standard deviation calculated for duplicates. The data for the no-cell control with aqueous U(VI)-dpaea were obtained in a replicate experiment. As the concentrations of aqueous U(VI)-dpaea were different, we display it in Figure S3C in the SI.

To evidence the implication of MHCs in the reduction of solid phase U(VI)-dpaea, the three strains WT, ΔOMC, and ΔOMCΔMtrA were incubated with solid-phase U(VI)-dpaea (the equivalent of 657 μM aqueous U(VI)) (Figure 1A), at an OD_600_ of 0.1. We chose to incubate the cells at a lower OD_600_ (as opposed to OD_600_ = 1 as above) to allow any differences in reduction rates among strains to stand out. The viable cell count decreased from 10^7^ to 10^5^ cells/mL after 48 h (Figure S3A). As mentioned for the Fe(III)-citrate experiment, the decrease in cell viability could be linked to substrate toxicity, but it may also be related to the resting cell experimental conditions, for which bacterial growth is not sustained. However, as the effect is observed for all three strains, it did not influence the interpretation of the results here. Both the WT and ΔOMC strains displayed rapid, and almost identical, reduction rates of solid-phase U(VI)-dpaea in the first 6 h, resulting in the accumulation of aqueous U evidenced by an increase from about 35 to 310 and 271 μM, respectively (Figure 1A). This rapid rise of aqueous U concentrations was followed by a second phase with slower kinetics from 24 to 72 h, up to 405 μM for WT incubations and up to 424 μM for ΔOMC incubations. Based on our previous study, we infer that the aqueous U released in the incubation supernatants corresponds to U(V)-dpaea.^21^ As for ΔOMCΔMtrA, it was impaired in solid-phase U(VI)-dpaea reduction as the aqueous U concentration remained stable over the first 24 h, oscillating between 34 and 51 μM (Figure 1A), and rose up to 67 μM after 72 h (a total of approximately 30 μM) but remained considerably lower than the concentration of U(V)-dpaea produced by ΔOMC or WT strains. Attempts to model the data with first-order kinetics were unsuccessful. Finally, the no-cell control exhibited no change in soluble U concentration over time (Figure S3C).

### Reduction of Aqueous U(VI)-dpaea and U(V)-dpaea by Mutant Strains

U(VI)-dpaea has a solubility of about 3 × 10^–5^M in aqueous medium at pH 7^21^ (depending on the U(VI)-dpaea batch), allowing discrimination of direct solid-phase reduction from solid U(VI)-dpaea dissolution followed by reduction of dissolved U(VI)-dpaea. To probe the contribution of each of the two mechanisms, reduction of the soluble fraction of U(VI)-dpaea was investigated. About 3 μM aqueous U(VI)-dpaea was incubated with the three strains WT, ΔOMC, and ΔOMCΔMtrA. We observed that all three strains reduced at least 85% of the 3 μM aqueous U(VI)-dpaea over a period of 72 h (Figure 1B). We have ruled out the possible adsorption of aqueous U(VI)-dpaea to the cells by analyzing both supernatant and cell pellet by IEC (Figure S4) and confirmed that the observed decrease in aqueous U(VI)-dpaea results in U(IV) precipitation in association with the cell pellet. A first-order kinetic model successfully reproduced the initial reduction rate, which was the highest for WT (0.0721 s^–1^) followed by ΔOMC (0.0371 s^–1^) and ΔOMCΔMtrA (0.0273 s^–1^) (Figure S5, Table S3). We additionally considered cell viability over the experimental time to confirm that active reduction occurred (Figure S3B). Moreover, no reduction was observed in the no-cell control displayed on Figure S3C.

**Figure 2:**
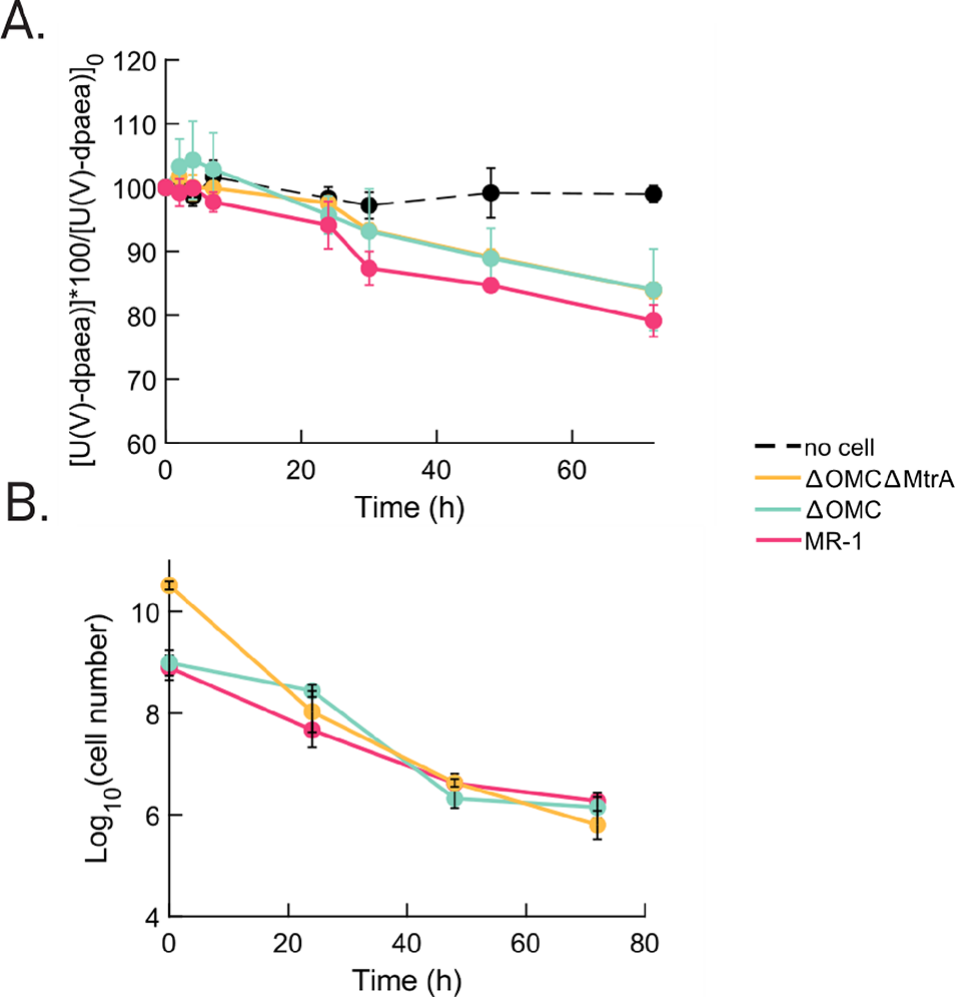
(A) Strains MR-1 (pink), ΔOMC (blue), and ΔOMCΔMtrA (yellow) incubated with 310 μM U(V)-dpaea. Aqueous U concentration in the filtrate (measured by ICP-MS) is shown here up to 72 h evidencing the decrease in aqueous U(V) due to the formation of insoluble U(IV). The no-cell control (black) shows no change in U concentration. The incubations were initially supplemented with 20 mM lactate as the electron donor, the cell density was OD_600_ = 1, and the pH value was 7.3. The results are displayed normalized in panel (A) as the starting concentration may vary slightly from one incubation to the other. (B) Cell viability of the WT and strains ΔOMC and ΔOMCΔMtrA over the experimental time in incubations with U(V)-dpaea. The cell viability was probed by counting colony forming units on LB agar plates. For both panels, the error bars correspond to the standard deviation calculated on quadruplicates per strain/no cell control.

In addition, the reduction of soluble U(V)-dpaea was investigated to better understand the role of MHCs. The three strains WT, ΔOMC, and ΔOMCΔMtrA were incubated with 310 μM U(V)-dpaea at a cell density of OD_600_ 1. We increased the cell density compared to the reactions with U(VI)-dpaea as the kinetics of reduction are known to be slower.^21^ After 72 h, the concentration of U(V)-dpaea showed a 21% decrease in the incubations with the WT and a 16% decrease in the incubations with ΔOMC and ΔOMCΔMtrA (Figure 2A). A first-order kinetic model of the reduction of U(V)-dpaea (Figure S6) evidenced similar initial rate constants (over the first 48 h), 0.0037 and 0.0036 s^–1^, respectively, for WT and ΔOMC, while ΔOMCΔMtrA exhibited a slightly slower rate, 0.0026 s^–1^ (Table S3). In contrast, in the no-cell control, the U(V)-dpaea concentration remained stable over the experimental time (Figure 2A), which ensures that U(V)-dpaea did not undergo spontaneous disproportionation. In addition, cell viability was followed over the experimental time to monitor active reduction (Figure 2B). Hence, we can conclude that the decrease in the U(V)-dpaea concentration observed in all cell incubations corresponds to enzymatic reduction of U(V)-dpaea to U(IV).

### Reduction with Purified MtrC

To further decipher the involvement of MHCs in the reduction of U(V)-dpaea, the reactions between U(V)-dpaea and the purified outer membrane MHC MtrC from *S. oneidensis* MR-1 (purification described in Text S7) were investigated. The functionality of reduced and dialyzed MtrC (procedure described in Text S8) was tested using 150 μM U(VI)-carbonate and U(VI)-citrate at a [U(VI)]/[MtrC] ratio of 1.15 and 0.9, respectively. Under these experimental conditions, we observed that MtrC reduced both substrates to a similar extent, 92 and 96.4 μM, respectively (corresponding to 60% and 64% of total U, respectively) after 30 s (data not shown). The results were obtained by probing the entire reaction mixture by IEC.

The reaction between U(V)-dpaea and either oxidized or reduced MtrC was monitored by IEC, to resolve the oxidation state of U after reaction. In addition, the redox state of the MtrC hemes was probed by UV–vis spectroscopy, in order to assess potential electron transfer. The no-protein control consisted of U(V)-dpaea only and thus, upon IEC treatment (with acid), is expected to yield 50% U(IV) and 50% U(VI), as a result of disproportionation. Any deviation from this ratio is attributable to reaction with MtrC.

**Figure 3:**
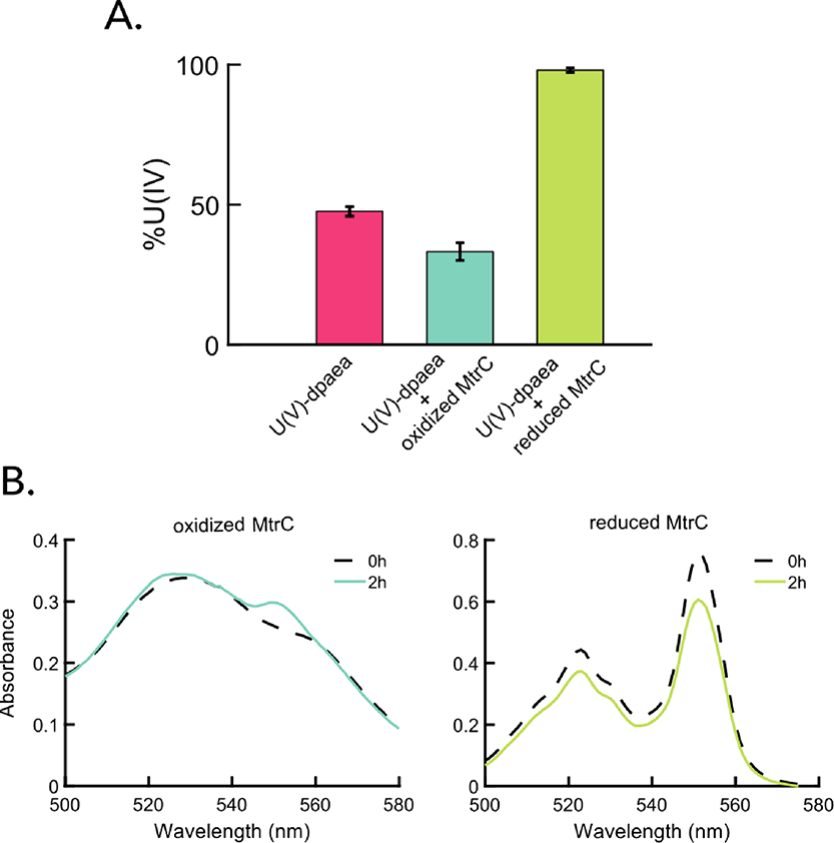
(A) Percentage of U(IV) obtained by ion exchange chromatography (IEC) after 2 min of reaction for the incubation of 150 μM U(V)-dpaea with either 150 μM of oxidized (light blue) or reduced (green) MtrC or no enzyme (pink). U(V)-dpaea in buffer A (pink) was used as a control for acid-induced disproportionation, which is expected for the IEC separation. IEC separation cannot directly identify U(V) because the samples are acidified prior to loading onto the column. As acid treatment is known to disproportionate uranyl(V), we expect that in the case of U(V)-dpaea, equal proportions of U(V) and U(IV) will be produced (result confirmed by U M_4_-edge HR-XANES^21^). The pH value of the enzymatic reactions between U(V)-dpaea and MtrC was set to 7.5. (B) UV–vis spectra of the hemes of oxidized MtrC (left) – all 10 heme Fe centers in Fe^3+^ configuration, and reduced MtrC (right) – all 10 heme Fe centers in Fe^2+^ configuration – before (dotted black) and after (solid line) reaction with U(V) dpaea for 2 h, recorded in anaerobic quartz cuvettes. The spectrum of oxidized MtrC displays a maximum in the region 500–600 nm at 530 nm, whereas the spectrum of reduced MtrC shows two peaks at 522 and 552 nm.

The reaction of U(V)-dpaea with reduced MtrC was probed to confirm that U(V)-dpaea can be transformed to U(IV) by a one-electron transfer. We noted the transformation of 118 μM U(V)-dpaea (corresponding to 98.8% of total U) to U(IV) species in 2 min (Figure 3A), and the hemes of MtrC were slightly oxidized (as determined after 2 h incubation, Figure 3B, right panel), suggesting that MtrC donated electrons to U(V)-dpaea. The possibility that U(V)-dpaea could be reduced by sodium dithionite left over from the MtrC reduction step was ruled out. The details of the test are provided in the experimental methods, and the results of the test are in Table S4.

In parallel, the reaction between U(V)-dpaea and oxidized MtrC was monitored. After 2 min, a decrease of 18 μM (14% of total U) in the U(IV) fraction (corresponding to an equal increase in the U(VI) fraction) was observed relative to the no-protein control (Figure 3A, Table S4), and the same result was observed after the reaction proceeded for 4 h (Figure S7, Table S5). Furthermore, MtrC hemes were slightly reduced. Indeed, the local absorption maximum at 530 nm (oxidized hemes) shifted to 522 nm and a second peak increased at 552 nm. These spectral features are characteristic of reduced hemes in MtrC (Figure 3B, left panel).

**Figure 4:**
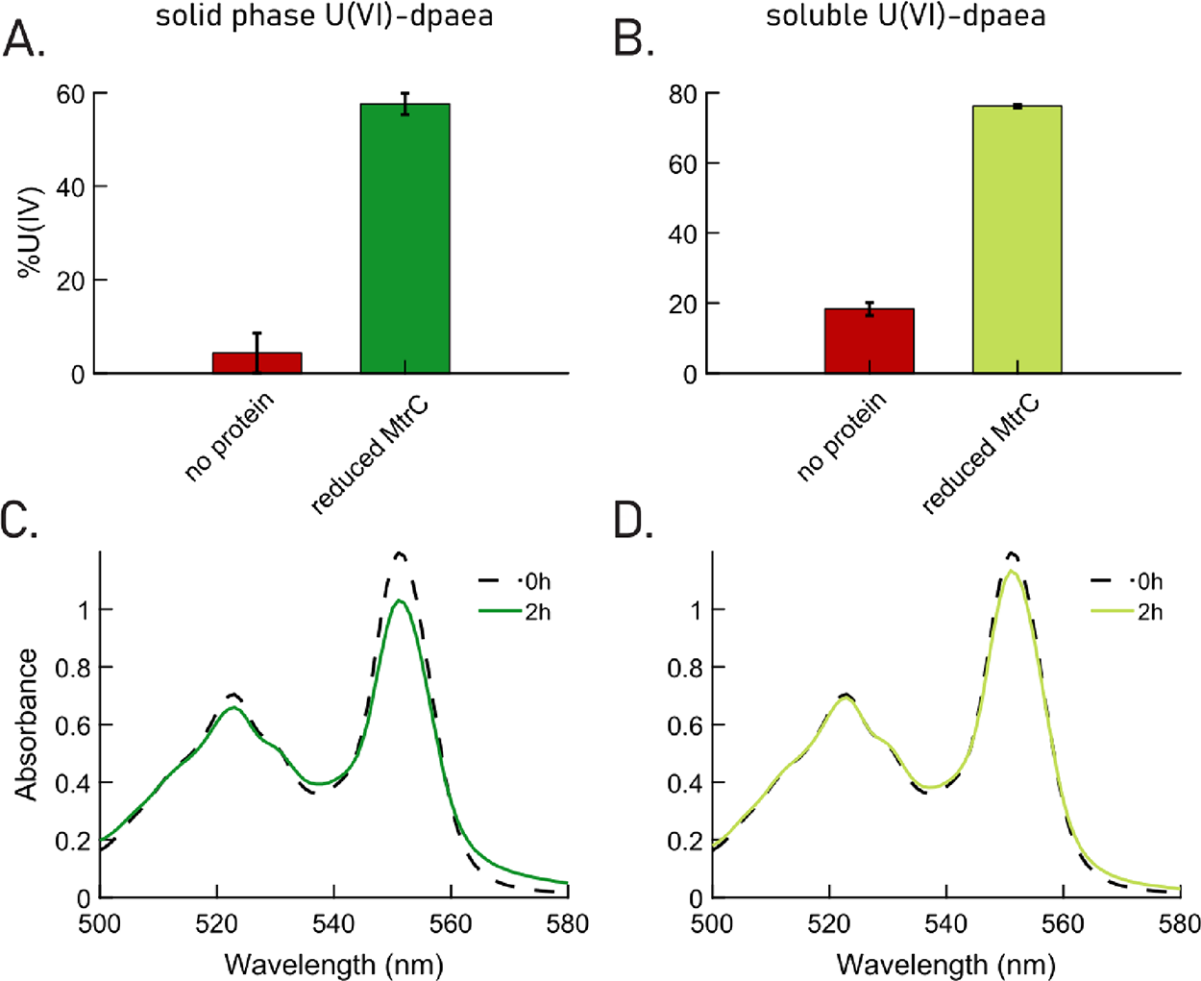
Percentage of U(IV) obtained by ion exchange chromatography after 2 min of reaction for (A) the incubation of 150 μM solid U(VI)-dpaea with reduced MtrC (green) or no enzyme (brown) or for (B) the incubation of 23 μM aqueous U(VI)-dpaea with reduced MtrC (green) or no enzyme (brown). The pH of the enzymatic reaction was set to 7.5. (C, D) UV–vis spectra of MtrC hemes before (dotted black) and after (solid U(VI)-dpaea: dark green; soluble U(VI)-dpaea: light green) reaction with U(VI)-dpaea.

Either solid or aqueous-phase U(VI)-dpaea was reacted with reduced MtrC (Text S9) and 88 μM solid-phase and 17.6 μM aqueous-phase U(VI) (corresponding to 60% and 80% total U) could be reduced by MtrC after 2 min, respectively (Figure 4A,B and Table S4). In addition, in both reactions, the hemes of MtrC underwent slight oxidation (Figure 4C,D), suggesting that electron transfer from the hemes occurred. Furthermore, the apparent U(IV) production rates observed for the reduction of U(VI)-dpaea substrates (0.19 and 0.11 μM s^–1^ for solid and aqueous phase, respectively) were slower than that for U(V)-dpaea (0.80 μM s^–1^) (Tables S6 and S7). However, the former entails a two-electron transfer from U(VI) to U(V), while the latter is only one electron transfer. Thus, the rates of reduction of U(V)-dpaea and U(VI)-dpaea by MtrC are not readily comparable.

## Discussion

We investigated the role of MHCs in the reduction of solid phase U(VI)-dpaea, aqueous U(VI)-dpaea, and aqueous U(V)-dpaea using either whole cells incubations of MR-1 WT, ΔOMC, and ΔOMCΔMtrA or the purified *c*-type cytochrome MtrC.

The two new constructs ΔOMC and ΔOMCΔMtrA were first characterized with respect to Fe(III) reduction to probe the expectation that solid-phase Fe(III) would not be reduced by either construct due to the involvement of outer membrane MHCs, while soluble Fe(III) (iron-citrate complex) would be reduced by both due to its expected diffusion into the periplasmic space. The results for ΔOMC confirmed that outer membrane MHCs are required for the reduction of ferrihydrite (Figure S1B). Edwards et al. have shown that in the MtrCAB complex in *Shewanella baltica* OS185, one heme of MtrA is solvent-exposed in the absence of MtrC. Potentially, in this scenario, MtrA could transfer electrons to extracellular electron acceptors.^38^ However, the results revealed that solid-phase Fe(III) was not reduced by the single solvent-exposed MtrA heme,^38^ presumed to be available in ΔOMC, likely due to inaccessibility of the rigid iron oxyhydroxide particles to the very surface of the cell. In addition, unexpected limited reduction of Fe(III)-citrate (Figure S1A) by both ΔOMC and ΔOMCΔMtrA was observed. Possibly, Fe(III)-citrate presumed to form a 1:1 or 1:2 aqueous complex may, in fact, form polynuclear Fe-citrate species (estimated at 72 Å^39^), too large to diffuse into the periplasm (size limit estimated at 13 kDa ≈ 54 Å^40^). Indeed, several studies pointed to the formation of polynuclear Fe(III) species in the presence of citrate at circumneutral pH, when the ratio of [Fe(III)]/[citrate] is close to stoichiometry.^39,41−44^ The polynuclear species may also be too large to allow full access to the one MtrA solvent-exposed heme, resulting in slower reduction of ΔOMC as compared to WT. Abiotic reduction of Fe(III)-citrate by lysed cells rather than by outer membrane MHCs was ruled out up until 24 h because there was no detectable reduction, and cell viability only started decreasing radically after 24 h (Figure S2A). Hence, these results suggest that the site of reduction for both forms of Fe(III) is the outer membrane and inform the interpretation of the results obtained for the reduction of uranium.

For solid-phase U(VI)-dpaea, we observed that, contrary to both ferrihydrite and Fe(III)-citrate, it is reduced by ΔOMC and WT at identical rates. Thus, for whole cells in the absence of MtrC, MtrA can readily transfer electrons to U(VI)-dpaea (Figure 1A). Furthermore, no first-order kinetic model fits the data, suggesting more complex dynamics than reduction of U(VI)-dpaea and release into solution of U(V)-dpaea. These results are reminiscent of the need to invoke dissolution followed by reduction as was previously reported for boltwoodite reduction.^34^ Hence, reduction of U(VI)-dpaea would progress predominantly via dissolution of solid U(VI)-dpaea followed by reduction of aqueous U(VI)-dpaea rather than by direct electron transfer to the solid phase. Indeed, the rates of reduction of solid U(VI)-dpaea by the WT and ΔOMC strains are comparable (Figure 1A); however, the particles of solid-phase U(VI)-dpaea may be too bulky to access the exposed heme of MtrA in ΔOMC. This latter hypothesis is supported by the impairment of ΔOMC in reducing ferrihydrite (Figure S1B), which suggests that ferrihydrite particles are too bulky to reach the solvent-exposed heme of MtrA. Furthermore, the solubility of ferrihydrite (2 × 10^–9^M^21^) is four orders of magnitude lower than that of solid-phase U(VI)-dpaea (3 × 10^–5^M^59^). The solubility comparison implies that the concentration of dissolved Fe(III) is too low to contribute to iron reduction; thus, direct electron transfer to solid Fe particles by outer membrane MHCs is the dominant mechanism. In contrast, dissolved U(VI)-dpaea could sustain the reduction of aqueous U(VI)-dpaea. As the equilibration of solid/aqueous phases is rapid, reduction of aqueous U(VI)-dpaea would drive the instantaneous dissolution of more solid phase.

The former mechanism is also supported (but not proven) by the comparison of reduction rates for aqueous U(VI)-dpaea by WT (0.0721 s^–1^), ΔOMC (0.0371 s^–1^), and ΔOMCΔMtrA (0.0273 s^–1^) strains (Figure S5), pointing to the predominant involvement of outer membrane MHCs in the reduction of aqueous U(VI)-dpaea. Periplasmic proteins contribute to aqueous U(VI)-dpaea reduction, only in the case for which MHCs are not present to reduce U(VI) prior to its diffusion into the periplasm (i.e., ΔOMCΔMtrA). This conclusion is further supported by the rapid reduction of aqueous U(VI)-dpaea by MtrC. Thus, the role of outer membrane MHCs, specifically MtrC, in the reduction of soluble U(VI) substrates is supported and confirms the observation of Marshall et al. on the reduction of aqueous U(VI)-citrate by MtrC.^18^

Thus, diffusion through the outer membrane and access to the pool of periplasmic MHCs is a possible mechanism of aqueous U(VI)-dpaea reduction, but its contribution to the overall reduction of solid-phase U(VI)-dpaea is limited. Furthermore, the involvement of outer membrane MHCs is supported by the fact that the purified outer membrane *c*-type cytochromes could rapidly reduce solid-phase U(VI)-dpaea in enzymatic reactions (Figure 4A). Altogether, these results point to a mechanism of solid-phase U(VI)-dpaea reduction consisting of dissolution and the reduction of aqueous U(VI)-dpaea being mediated primarily by outer membrane MHCs.

Additionally, the reduction of U(V)-dpaea by strains WT, ΔOMC, and ΔOMCΔMtrA was probed. The first two strains exhibited identically slow reduction rates (∼0.0037 s^–1^), while ΔOMCΔMtrA was slower (0.0026 s^–1^) (Figure 2A, Table S3). We interpret these data as pointing to the dominant role of outer membrane MHCs in the reduction here as well. Indeed, the same rate for WT and ΔOMC results from the fact that the kinetics of enzymatic reduction are slow enough that diffusion to access the solvent-exposed heme of MtrA in ΔOMCΔMtrA is faster than the reduction itself. This is also why reduction by strain ΔOMCΔMtrA is only ∼30% slower than that by WT and ΔOMC (whereas it is 60% slower for U(VI)-dpaea). Nonetheless, periplasmic MHCs must also be involved to a lesser extent, particularly in the absence of outer membrane MHCs, to account for the reduction by strain ΔOMCΔMtrA.

The proteins that could reduce aqueous U(V)-dpaea (this also extends to aqueous U(VI)-dpaea mentioned above) in the periplasm include the periplasmic tetraheme MHCs STC and FccA and the inner membrane tetraheme MHC CymA, which mediates electron transfer between the quinone pool and the periplasm. STC and FccA are the most abundant MHCs in the periplasm of *S. oneidensis* MR-1.^45^ Previous work has demonstrated the reduction of various Fe(III)-ligand complexes by STC^46^ and CymA.^47^ Firer-Sherwood et al. have measured and compared the electrochemical potentials of MtrC, OmcA, MtrA, STC, and CymA from *S. oneidensis* MR-1,^48^ evidencing the fact that STC and CymA have overlapping redox potentials with MtrC. Finally, Marshall et al. have demonstrated the reduction of U(VI) and periplasmic accumulation of U(IV) for two deletion mutants lacking MtrC or MtrC/OmcA.^18^

Moreover, the contribution of riboflavins to the reduction mechanism of solid-phase U(VI)-dpaea and aqueous U(V)-dpaea has been considered. Reduction experiments with a mutant strain lacking the riboflavin transporter Δ*bfe* (Text S10 and Tables S8 and S9), hence impaired in its ability to export flavin to the extracellular medium, showed no impairment in solid U(VI)-dpaea nor U(V)-dpaea reduction compared to the WT (Figure S8A,B, respectively). This observation suggests that flavin-mediated electron transfer may not be a significant mechanism for the reduction for either solid U(VI)-dpaea or aqueous U(V)-dpaea, confirming the results published by Cherkouk et al.^16^ However, these results should be confirmed with a mutant lacking all membrane MHCs and MtrA as well as the flavin transporter.

Additionally, *in vitro* experiments with the purified MHC MtrC were performed to investigate the mechanism of reduction of U(V)-dpaea by the enzyme. The two possibilities were either reduction of U(V)-dpaea via a one-electron transfer or the disproportionation of U(V) to U(VI) and U(IV) followed by the reduction of U(VI)-dpaea to U(V)-dpaea. We prepared MtrC in either its oxidized state, i.e., when all hemes are in the Fe^3+^ form, or in its reduced state, i.e., all hemes are in the Fe^2+^ form. Deconvoluting the direct reduction of U(V)-dpaea by MtrC vs disproportionation of U(V)-dpaea to U(VI) and U(IV) requires considering the reduction of U(VI) by reduced MtrC as well as the oxidation of U(IV) by oxidized MtrC. In addition, it is important to probe whether the interaction between MtrC and U(V)-dpaea does not, in itself, trigger disproportionation of U(V)-dpaea. The reaction between oxidized MtrC and U(V)-dpaea led to partial reduction of MtrC. Thus, the interaction between U(V)-dpaea and MtrC does not lead to disproportionation.

Additionally, the reduction of U(V)-dpaea by reduced MtrC proceeded rapidly and resulted in the oxidation of MtrC, supporting one-electron transfer (Figure 3A,B, right panel). However, to exclude the possibility that disproportionation followed by U(VI) reduction occurs, we probed the reduction of aqueous and solid-phase U(VI)-dpaea by reduced MtrC (Figure 4). While comparing the formation rates of U(IV) from U(VI)-dpaea and U(V)-dpaea is not a rigorous exercise (as one is a one- and the other a two-electron transfer), we can still show that the rates are not compatible with the mechanism of reduction being U(V) disproportionation followed by U(VI) reduction to U(IV) as the latter is much slower than the reduction of U(V)-dpaea to U(IV) (Table S7). Rather, U(V)-dpaea is reduced to U(IV) via a one-electron transfer. In addition, to rule out the scenario of disproportionation followed by re-oxidation of the formed U(IV) species by oxidized MtrC, the reaction between U(IV)-citrate and oxidized MtrC was investigated. U(IV)-citrate was not altered by oxidized MtrC (Figure S9A,B), confirming that disproportionation of U(V)-dpaea followed by oxidation of U(IV) is not a viable mechanism. Therefore, as the reduction of U(V)-dpaea is much faster than that of aqueous U(VI)-dpaea and the oxidation of U(IV) by oxidized MtrC does not proceed, our results point to a direct reduction mechanism of U(V)-dpaea by reduced MtrC.

## Environmental Relevance

**Figure 5:**
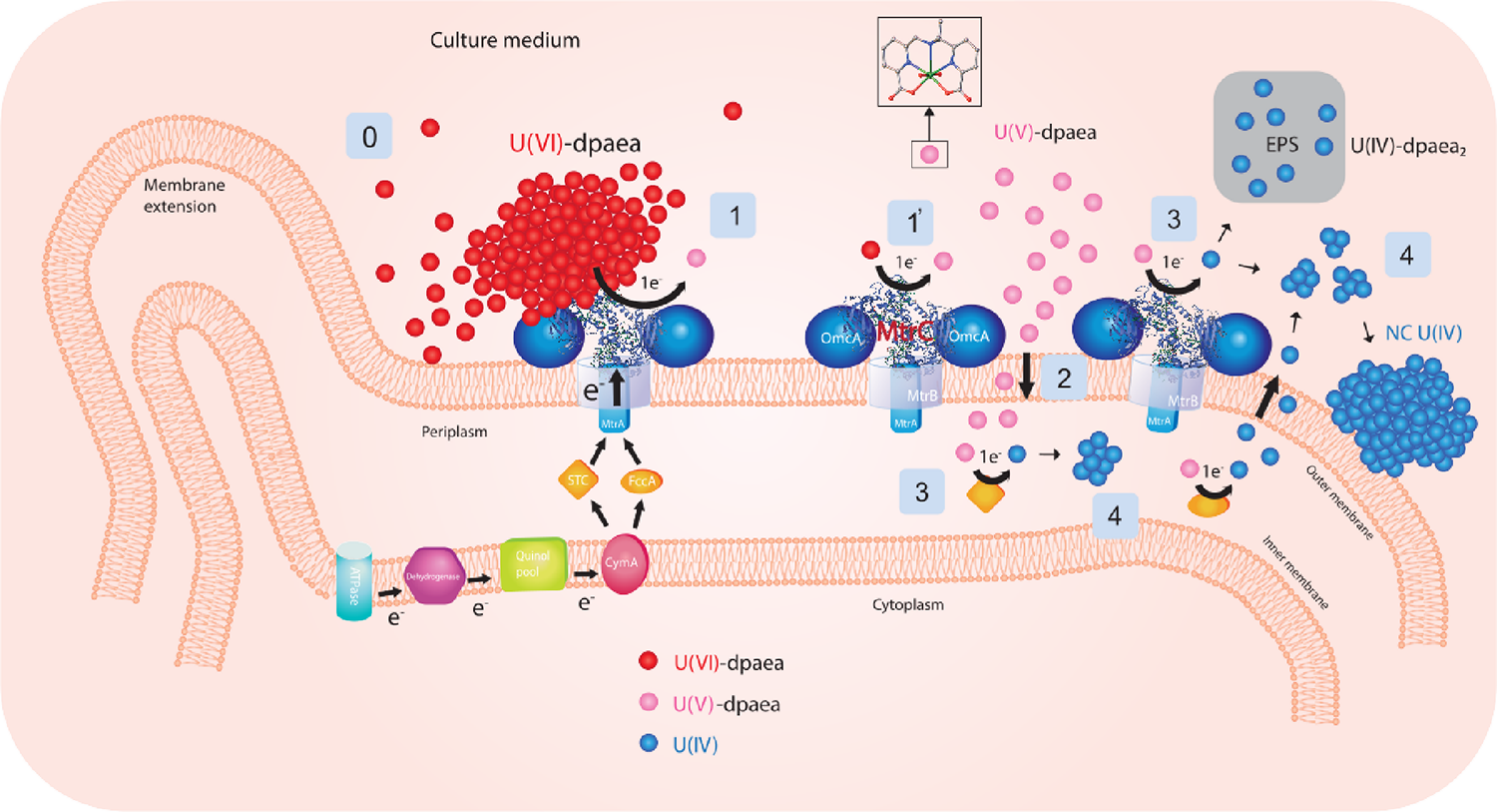
Summary of the study findings depicting the mechanism of reduction of solid-phase U(VI)-dpaea (in red) and aqueous U(V)-dpaea (in pink) to amorphous U(IV) and organic complexes of U(IV) (in blue). Step 0 corresponds to the limited dissolution of U(VI)-dpaea. Step 1 depicts the reduction of solid-phase U(VI)-dpaea by direct contact with the outer membrane MHC (likely limited). Step 1′ shows U(VI)-dpaea reduction via the dissolved fraction (likely dominant). Step 2 represents the potential migration of U(V)-dpaea into the cell periplasm. Step 3 shows the reduction of U(V)-dpaea by MtrC on the outer membrane but also by the periplasmic MHC. Step 4 describes the formation of U(IV) products, non-crystalline in association with the cells and monomeric associated with the extracellular polymeric substance.

This study provides direct evidence that U(V)-dpaea can be biologically reduced to U(IV) species by MHCs and shows that outer membrane MHCs are the primary reductases for solid-phase U(VI)-dpaea. In addition, we demonstrated that solid U(VI)-dpaea reduction occurs via dissolution followed by reduction of the dissolved aqueous U(VI)-dpaea. The results are summarized Figure 5.

We chose to focus our study on the synthetic ligand dpaea. We harnessed its unique property of forming a stable U(V) complex in aqueous solution at pH 7 to investigate the enzymatic reduction of U(V) and provide insights into the molecular mechanisms of U(V) bioreduction. These results provide an impetus to study other ligands that could stabilize U(V). Indeed, dpaea belongs to the class of aminocarboxylate ligands, comprising environmentally relevant ligands such as dpa (produced by bacterial endospores^22,23^) or EDTA and NTA (from anthropogenic contamination^24−28^). The structures of the U-dpaea, U-dpa, U-EDTA, and U-NTA are very similar as all these ligands occupy the equatorial plane, surrounding the U atom. Hence, these ligands are promising candidates for the stabilization of U(V), and recent studies have suggested that dpa could stabilize U(V) in aqueous solution.^49,50^

Very little is known about the distribution of U(V) in the environment, even though it was identified in the matrix of a 1.6 billion year-old hematite by Ilton et al.,^51^ opening the door to a quest for U(V) in the environment. Indeed, U(V) chemistry and its resulting stability is dependent on the local chemical environment: available organic carbon, presence of mineral phases, physical state of U (solid/aqueous), pH, electrochemical potential, and O_2_ levels. Up until now, laboratory experiments have demonstrated that abiotic U(V) can be stabilized within iron oxides such as hematite,^52^ magnetite,^53−55^ goethite,^56^ and green rust,^57^ and that biological U(V) can be stabilized by appropriate organic ligands,^21,49^ precluding disproportionation. Hence, the environmental occurrence and persistence of biologically generated U(V) remains to be investigated, but this study points to the types of systems (e.g., those with abundant complex ligands) in which U(V) could persist.

While solid-phase U(VI)-dpaea is not directly environmentally relevant, it allows the investigation of solid-phase U(VI) reduction. The mechanism of reduction of solid-phase U(VI), whether direct electron transfer or dissolution followed by reduction, is relevant to U(VI) minerals for instance at mining sites^58^ (e.g., U(VI)-phosphate phases) and to the long-term sequestration of U(VI) in these minerals. This study shows that dissolution followed by the reduction is the most likely mechanism for U(VI) minerals.

## Supporting Information Available

Additional experimental details on the deletion mutant constructs, the bacterial growth conditions, the uranium quantification method, the synthesis of ferrihydrite and following biological reduction experiments, purification protocol of MtrC, reduction protocol of MtrC and its reaction with U(VI)-dpaea substrates; figures for Fe(III) reduction experiments, cell viability in the presence of either Fe or U, U oxidation state in whole cell incubations with aqueous U(VI)-dpaea, initial reaction rate of whole cells incubated with U(VI)-dpaea or U(V)-dpaea substrates, additional data on the U(V)-dpaea reduction experiment with MtrC, results of the incubation of the flavin transporter mutant (Δ*bfe*) incubated with U(VI)-dpaea or U(V)-dpaea, and reaction of oxidized MtrC with U(IV)-citrate; tables of the primers used for the deletion mutant constructs, first-order kinetic constants for U(VI)-dpaea or U(V)-dpaea reduction by *S. oneidensis*, rates of reaction of U species with purified MtrC, and the riboflavin profile of the flavin transporter mutant (Δ*bfe*) incubated with U(VI)-dpaea or U(V)-dpaea (PDF)
