# Supplementary material for: Mechanism of Reduction of Aqueous U(V)-dpaea and Solid-Phase U(VI)-dpaea Complexes: The Role of Multiheme c-Type Cytochromes
Source: Environ Sci Technol. 2023 May 3;57(19):7537–46. doi: 10.1021/acs.est.3c00666 (PMC10193582; doi:10.1021/acs.est.3c00666)
Supplement: Supplementary file 1 — es3c00666_si_001.pdf [file es3c00666_si_001.pdf]

Supplementary information

Mechanism of reduction of aqueous U(V)-dpaea and solid-phase U(VI)-dpaea complexes: the role of multiheme c-type cytochromes

Margaux Molinas[1]*, Karin Lederballe Meibom [1], Radmila Faizova[2], Marinella Mazzanti[2], Rizlan Bernier-Latmani[1]*.

[1] Environmental Microbiology Laboratory, and [2] Group of Coordination Chemistry, Ecole Polytechnique Fédérale de Lausanne (EPFL), Lausanne, 1015, Switzerland

*Corresponding author
Rizlan Bernier-Latmani
rizlan.bernier-latmani@epfl.ch

Margaux Molinas
margaux.molinas@gmail.com

26 pages: 10 text sections, 9 figures, 9 tables, and references

This supporting information contains:

Text S1. Description of the *mtrC/omcA/mtrF* deletion mutant.
Text S2. Description of the *mtrC/omcA/mtrF/mtrA* deletion mutant.
Text S3. Strain and growth conditions.
Text S4. Uranium quantification.
Text S5. Preparation of ferrihydrite and Fe(III)-citrate.
Text S6. Reduction experiments with ferrihydrite and Fe(III)-citrate.
Text S7. Protein purification.
Text S8. Reduction of MtrC
Text S9. Reaction of MtrC with solid phase U(VI)-dpaea and aqueous U(VI)-dpaea
Text S10. Description of the *bfe* deletion mutant

Figure S1: Fe(II) concentration measured by ferrozine test in the incubation supernatants of MR-1 (pink), ΔOMC (blue), ΔOMCΔMtrA (yellow) and a no-cell control (black) from 0 to 48h of incubation with A. 2000µM Fe(III)-citrate, B. 2000µM ferrihydrite.
Figure S2. Cell viability of MR-1(pink dots), ΔOMC (blue dots) and ΔOMCΔMtrA (yellow dots) over the experimental time in incubations with A. Fe(III)-citrate, B. Ferrihydrite.
Figure S3. Cell viability of MR-1 (pink dots), ΔOMC (blue dots) and ΔOMCΔMtrA (yellow dots) over the experimental time in incubations with A. solid phase U(VI)-dpaea and B. aqueous U(VI)-dpaea. C. Concentration of U(VI)-dpaea in solution for a no-cell control (black) and MR-1 (pink).
Figure S4: U oxidation state in the solid phase (cell pellet) and in the aqueous phase (supernatant) of A. incubations with *S. oneidensis* MR-1; B. and no-cell controls, incubated with 20µM aqueous U(VI)-dpaea, at pH 7.3, OD_{600} =1.
Figure S5. Initial rate of reaction for aqueous U(VI)-dpaea incubated with WT *S. oneidensis* MR-1 (pink), ΔOMC (blue) and ΔOMCΔMtrA (yellow). The data are only considered for the first 7h of the experiment.

Figure S6. Initial rate of reaction of aqueous U(V)-dpaea incubated with WT *S. oneidensis* MR-1 (pink), ΔOMC (blue) and ΔOMCΔMtrA (yellow). The data are only considered for the first 48h of the experiment.
Figure S7. Percentage of U(IV) obtained by ion exchange chromatography of the reaction between U(V)-dpaea and either oxidized (blue) or reduced (green) MtrC after 20s, 2h and 4h of reaction. U(V)-dpaea (pink) was used as a control to ensure that no spontaneous disproportionation occurred during the experimental time.
Figure S8. Incubation of the WT MR-1 strain (pink) and a mutant strain of MR-1 lacking the flavin transporter system (dark blue) with A. solid U(VI)-dpaea, B. aqueous U(V)-dpaea.
Figure S9. A. Percentage of U(IV) obtained by ion exchange chromatography of the reaction between 100μM U(IV)-citrate and 100μM oxidized MtrC (light blue) after 2 min of reaction. U(IV)-citrate in buffer A (dark blue) was used as a control for the U oxidation state. **B.** UV-vis spectra of the hemes of MtrC before (dotted black) and after (light blue) reaction (2 hours) with U(IV) citrate.

Table S1. Primers used for PCR and sequencing for the Δ*mtrC/omcA/mtrF* construct.
Table S2. Primers used for PCR and sequencing for the Δ*mtrC/omcA/mtrF/mtrA* construct.
Table S3. First-order kinetic constants for incubations of aqueous U(VI)-dpaea or U(V)-dpaea with WT, ΔOMC and ΔMtrA *S. oneidensis* MR-1 strains.
Table S4. Summary of the experimental results describing the reactions of U(V)-dpaea with either oxidized or reduced MtrC after 2 min, and also with the dialysis control (no protein control) obtained by dialyzing the amount of sodium dithionite used to reduce MtrC. Additionally, the reaction of U(IV)-citrate with oxidized MtrC is reported. We also included the results from the reaction between reduced MtrC and solid or soluble U(VI)-dpaea.
Table S5. Summary of the experimental results corresponding to Figure S7 describing the reactions of U(V)-dpaea with either oxidized or reduced MtrC over 4h.
Table S6. U(VI) and U(IV) concentrations and percentage obtained by ion exchange chromatography (two left columns) and proposed derived U(IV) and U(V) concentrations and percentage (two right columns) in the reduction of solid and aqueous U(VI)-dpaea, and aqueous U(V)-dpaea by purified and reduced MtrC.
Table S7. Apparent formation rates of U(IV) species upon reduction of solid U(VI)-dpaea, aqueous U(VI)-dpaea, and aqueous U(V)-dpaea by purified and reduced MtrC.
Table S8. Primers used for PCR and sequencing for the Δ*bfe* construct.
Table S9. Riboflavins profile over 72h measured by HPLC for both the WT and Δ*bfe* when incubated with 4mM U(VI)-dpaea.

Text S1. Description of the *mtrC/omcA/mtrF* deletion mutant

Regions flanking *mtrF* (SO_1780) in *Shewanella oneidensis* Δ*omcA*Δ*mtrC* were amplified by PCR with primers mtrF_5'O/mtrF_5'I and mtrF_3'I/mtrF_3'O(DD) (Table S 1), fused by overlap extension PCR and cloned into suicide plasmid pMQS[1]. The resulting plasmid, pMQS-mtrF(DD), was introduced into *Shewanella oneidensis* Δ*omcA*Δ*mtrC* by conjugation from *E. coli* strain WM3064. Colonies with single crossover plasmid insertions were selected on LB agar plates containing kanamycin, purified once on agar plates with kanamycin and resistant colonies were subsequently grown overnight in LB (containing no NaCl) without antibiotic. Double crossover mutants were selected on LB agar plates (containing no NaCl) supplemented with 10% sucrose. Sucrose resistant and kanamycin sensitive colonies were checked by colony PCR for gene deletion using primers flanking the deleted region (mtrF_FO + mtrF_RO(DD)) (Table S1). Selected clones were purified, genomic DNA isolated, and the region containing the deleted gene was amplified by PCR and the deletion verified by Sanger sequencing (Table S1). Henceforth, for simplicity, the Δ*mtrC/omcA/mtrF* deletion mutant will be referred as ΔOMC. 'OMC' stands for outer-membrane *c*-type cytochromes.

Text S2. Description of the *mtrC/omcA/mtrF/mtrA* deletion mutant

Regions flanking *mtrA* (SO_1777) in *Shewanella oneidensis* Δ*mtrC/omcA/mtrF* were amplified by PCR with primers mtrA_5'O/mtrA_5'I and mtrA_3'I/mtrA_3'O(Table S2), fused by overlap extension PCR and cloned into suicide plasmid pMQS[1]. The resulting plasmid, pMQS-mtrA, was introduced into *Shewanella oneidensis* Δ*mtrC/omcA/mtrF* by conjugation from *E. coli* strain WM3064. Colonies with single crossover plasmid insertions were selected on LB agar plates containing kanamycin, purified once on agar plates with kanamycin and resistant colonies were subsequently grown overnight in LB (containing no NaCl) without antibiotic. Double crossover mutants were selected on LB agar plates (containing no NaCl)

supplemented with 10% sucrose. Sucrose resistant and kanamycin sensitive colonies were checked by colony PCR for gene deletion using primers flanking the deleted region (mtrA_FO + mtrA_RO) (Table S2). Selected clones were purified, genomic DNA isolated, and the region containing the deleted gene was amplified by PCR and the deletion verified by Sanger sequencing (Table S2). Henceforth, for simplicity, the Δ*mtrC*/*omcA*/*mtrF*/*mtrA* deletion mutant will be referred as ΔOMCΔMtrA.

Text S3. Strain and growth conditions

In addition to WT *S. oneidensis* MR-1, this study also includes the newly generated mutants, ΔOMC and ΔOMCΔMtrA. The three strains were first incubated in a liquid pre-culture started from a frozen stock (-80°C) and grown overnight in Luria-Bertani (LB) medium at 30°C in a shaking incubator (140 rpm). An aliquot of this pre-culture was further inoculated in fresh LB with a starting OD_{600} of 0.1. When the culture reached an OD_{600} of 2 (mid- to late-exponential phase), the cells were harvested by centrifugation at 9,610 ×g for 10 minutes at room temperature (Avanti J-26 XP, Beckman, Brea, California, US), and washed three times anoxically with modified Widdel low phosphate (WLP) medium at pH 7.3, prior to amendment with U. The modified WLP medium lacked bicarbonate and phosphate to prevent their complexation of U[2].

Text S4. Uranium quantification

U concentration was measured by Inductively Coupled Plasma Mass Spectrometry (ICP-MS 7900, Agilent, Santa Clara, California, US) for both filtered supernatants and samples eluted from the ion exchange chromatography test. Dilutions to a range of 1 to 10 ppb of U were performed in a matrix of 1% HNO_3 prior to analysis. All samples were measured in technical duplicates.

Text S5: Preparation of ferrihydrite and Fe(III)-citrate

Fresh ferrihydrite was prepared by titrating 0.5M Fe(III)Cl_3 with 1M NaOH until a neutral pH value. After 30 minutes of equilibration, the precipitate was centrifuged and washed 5 times with deionized water (8,000 g, 10 min). A Fe(III)-citrate stock solution was prepared by dissolving Fe(III)-citrate (M_w = 245.95g/mol) in deionized water and adjusting the pH value to 7 with 1M NaOH. The obtained solution was filter-sterilized (0.2 μm). The total iron concentration in both the ferrihydrite suspension and Fe(III)-citrate solution was measured by ICP-OES after digestion of a ferrihydrite aliquot or dilution of Fe(III)-citrate in 4.5M HCl. The suspension and solution were both stored at 4°C in the dark.

Text S6: Reduction experiments with ferrihydrite and Fe(III)-citrate

All experiments described in the following paragraph were performed inside a nitrogen-atmosphere anaerobic chamber (MBraun, Germany), with O_2 < 0.1 ppm. Prior to incubation, *S. oneidensis* MR-1 WT, ΔOMC and ΔOMCΔMtrA cells were prepared as described in text S3. They were incubated, in addition to a no-cell control, under non-growth conditions in a modified WLP medium[2] supplemented with 20 mM lactate, the electron donor, and either 2 mM anoxic ferrihydrite or Fe(III)-citrate as electron acceptors. The starting OD_{600} of the incubations was calculated to be 1. The incubations were maintained in the dark at room temperature, inside the anaerobic chamber. Fe(II) was measured by the ferrozine assay and cell viability was evaluated by streaking an aliquot of culture on LB agar plates. Each experimental condition was run in duplicate.

Text S7. Protein purification.

Recombinant soluble MtrC was expressed in the double mutant Δ*omcA*Δ*mtrC* strain of *S. oneidensis* MR-1 LS331[4], kindly provided by Liang Shi. 5-10 L cultures were grown at 30ºC, and induced with 0.1 mM L-arabinose. Culture was collected by centrifugation after overnight growth @ 5000xg for 30 minutes. The clarified supernatant was loaded on a combination of 5 5 mL HisExcel resins at 5-6 mL/minute, and the protein was eluted with 40 mM imidazole and

150 mM NaCl. The flow-through was pooled, concentrated to 5 mL and loaded on to a Superdex 200 16/600, equilibrated with 150 mM NaCl and 20 mM HEPES at pH 7.5. Peak fractions were collected, pooled and concentrated. Following elution and concentration, purified MtrC migrated as a single band on an SDS-PAGE gel with an apparent mass of 75 kDa. The protein concentration was evaluated by bicinchoninic acid assay (Pierce™ BCA Protein Assay Kit, ThermoFisher Scientific, Waltham MA USA).

Text S8: Reduction of MtrC

In an MBraun glovebox, purified MtrC (protocol described text S8) was reduced using sodium dithionite ($Na_2S_2O_4$). Sodium dithionite was added gradually, until the hemes were fully reduced. Their redox status was monitored by UV-vis spectrophotometry (UV-2501P, Shimadzu, Kyoto Japan) at a wavelength range of 500-580 nm. An anaerobic quartz cuvette (Msscientific, Berlin, Germany) was used for this purpose. Reduced MtrC is characterized by two peaks at 522 nm and 552 nm (β and α Soret absorption peaks), whereas oxidized MtrC displays a maximum at 530 nm in this spectral region. In order to remove the potential excess of sodium dithionite, which could react with U, the reduced protein was dialyzed for about 18h using dialysis cassettes (Side-A-Lizer ®, ThermoFisher Scientific, Waltham MA USA) in the glovebox. The buffer (buffer A) used was composed of 100 mM HEPES and 50 mM NaCl, and the pH was adjusted to a value of 7.5. The redox status of the hemes was probed after dialysis in order to ensure that they were still fully reduced. The concentration was measured again with the Bicinchoninic acid (BCA) protein assay. To ensure that dialysis effectively removed excess sodium dithionite from the protein solution, sodium dithionite was prepared in buffer A at the same initial concentration as that used to reduce the cytochromes. The solution was dialyzed following the method described above and post-recovery, it was reacted with U(V)-dpaea. We did not observe reduction of U(V)-dpaea, suggesting that the dialysis time was sufficiently long for the complete diffusive removal of the reducing agent. This robustness

of the dialysis treatment was demonstrated by the reproducibility of the rate of reduction of U(V)-dpaea by reduced MtrC.

Text S9: Reaction of MtrC with solid phase U(VI)-dpaea and aqueous U(VI)-dpaea

In the glovebox, four reactions were initiated as follows: (i) solid U(VI)-dpaea in buffer A; (ii) solid U(VI)-dpaea in buffer A with reduced MtrC; (iii) aqueous U(VI)-dpaea in buffer A; (iv) aqueous phase U(VI)-dpaea in buffer A with reduced MtrC. Reactions (i) and (iii) served to control the initial oxidation state of U. Solid U(VI)-dpaea and protein solutions were prepared at a starting concentration of 300 μM. The initial U concentration in the solution of aqueous U(VI)-dpaea was measured to be 23 μM. The reactions were initiated by mixing equal volumes of U and MtrC. Timepoints were collected by removing an aliquot from the reaction mixture, and immediately loading it onto IEC resins to separate U(VI) from U(IV), as done previously[2]. The heme redox status was probed before and after the reaction by UV-vis spectrophotometry to evaluate how they were influenced by U. Both U(IV) and U(VI) fractions were quantified by ICP-MS.

Text S10. Description of the *bfe* deletion mutant

Regions flanking bfe (SO_0702) in *Shewanella oneidensis* MR-1 were amplified by PCR with primers bfe_5'O/bfe_5'I and bfe_3'I/bfe_3'O (Table S9), fused by overlap extension PCR and cloned into suicide plasmid pMQS as a KpnI-BamHI fragment[5]. The resulting plasmid, pMQS-bfe, was introduced into *Shewanella oneidensis* MR-1 by conjugation from *E. coli* strain WM3064. Colonies with single crossover plasmid insertions were selected on LB agar plates containing kanamycin, purified once on agar plates with kanamycin and resistant colonies were subsequently grown overnight in LB (containing no NaCl) without antibiotic. Double crossover mutants were selected on LB agar plates (containing no NaCl) supplemented with 10% sucrose. Sucrose resistant and kanamycin sensitive colonies were checked by colony PCR for gene deletion using primers flanking the deleted region (bfe_FO

+ bfe_RO) (Table S9). Selected clones were purified, genomic DNA isolated, and the region containing the deleted gene was amplified by PCR and the deletion verified by Sanger sequencing (Table S9). The flavin secretion profile was compared to that of the WT MR-1 by analyzing aliquots of both the *bfe* deletion mutant (Δ*bfe*) and the WT incubations with 4mM U(VI)-dpaea by, in triplicate, after 0h, 24h, 72h. HPLC was used to quantify the flavins following the protocol described by D. E. Ross al.[6]. Only riboflavins were detected and to a lesser extent in the Δ*bfe* strain over 72h, confirming that the strain is less efficient in the transport of flavins to the extracellular medium (Table S10).

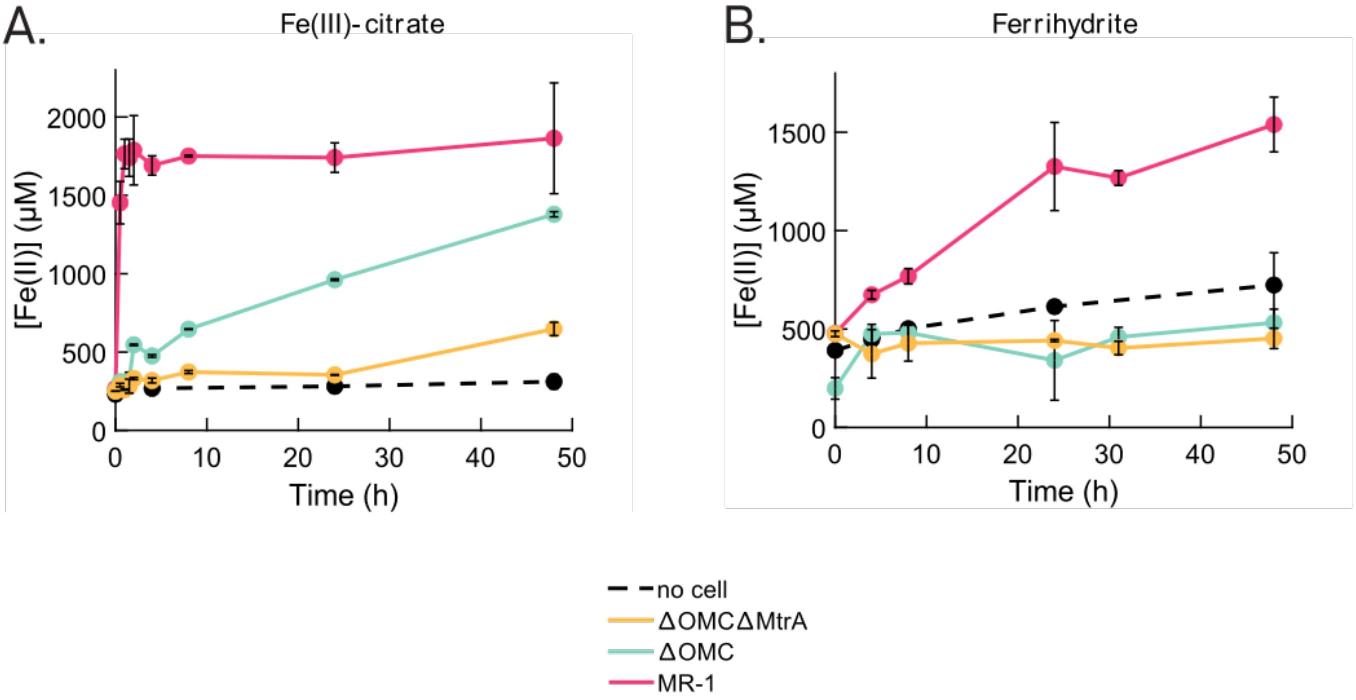

Figure S1: Fe(II) concentration measured by ferrozine test in the incubation supernatants of MR-1 (pink), ΔOMC (blue), ΔOMCΔMtrA (yellow) and a no-cell control (black) from 0 to 48h with **A.** 2,000μM Fe(III)-citrate, **B.** 2,000μM ferrihydrite. The incubations were initially supplemented with 20mM lactate as the electron donor, the cell density was set to OD_{600} =1, and the pH of the experiment was 7.3. The error bars correspond to the range calculated for duplicates per strain/no cell control.

The observed increase in Fe(II) concentration for the no cell control in the ferrihydrite experiment (B.) is due to potential contamination of the starting ferrihydrite material, as it is not strictly sterile. Here, the reduction of aqueous Fe(III)-citrate (A.) and solid ferrihydrite (B.) is investigated by MR-1 strain and two engineered strains of MR-1. ΔOMC lacks outer-membrane MHCs MtrC, OmcA and MtrF, and ΔOMCΔMtrA lacks in addition the transmembrane MHC MtrA. If reduction occurs, Fe(II) is measurable in the incubation supernatants. For both substrates, MR-1 (pink) reduces Fe(III), whereas, the engineered strains ΔOMC (blue), ΔOMCΔMtrA (yellow) showed impairment in Fe(III) reduction. For Fe(III)-

citrate, the potential large Fe clusters formed may prevent Fe(III) to access MtrA in ΔOMC, or diffuse in the periplasm in ΔOMCΔMtrA. Regarding ferrihydrite, the outer-membrane MHCs are clearly required to access the solid phase Fe(III). These results helped in the characterization of ΔOMC and ΔOMCΔMtrA deletion mutant strains, and confirm their altered functioning.

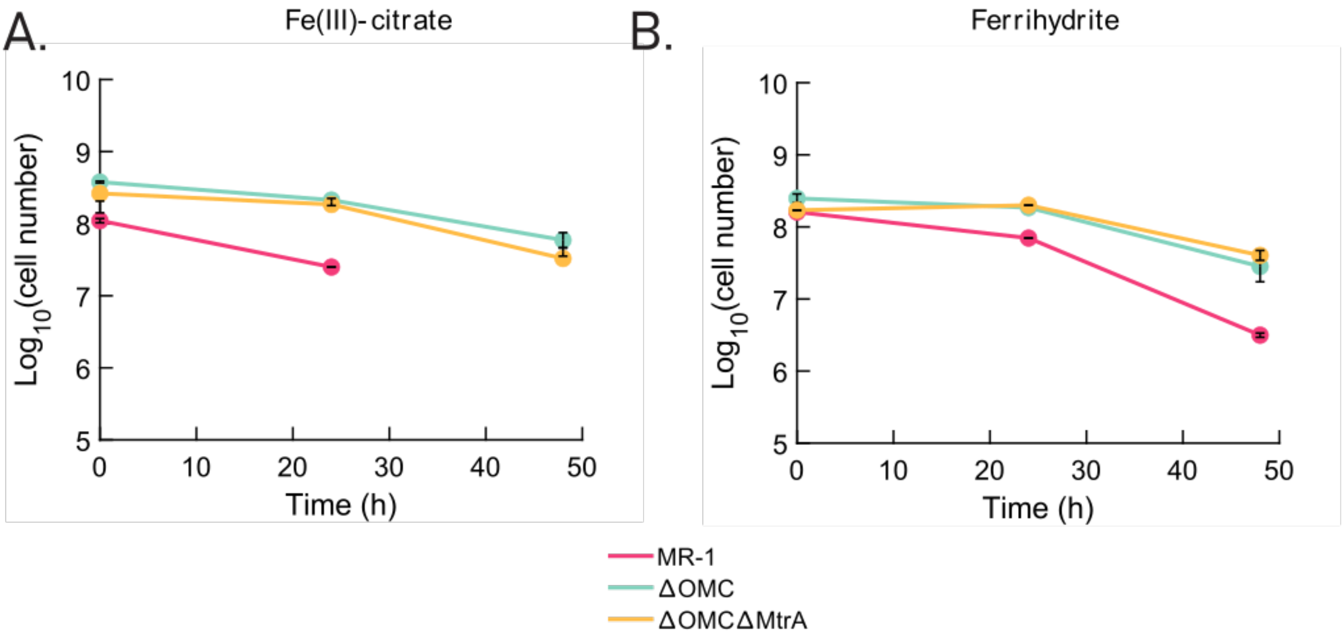

Figure S2: Cell viability of MR-1(pink dots), ΔOMC (blue dots) and ΔOMCΔMtrA (yellow dots) over the experimental time in incubations with A. Fe(III)-citrate, B. Ferrihydrite.

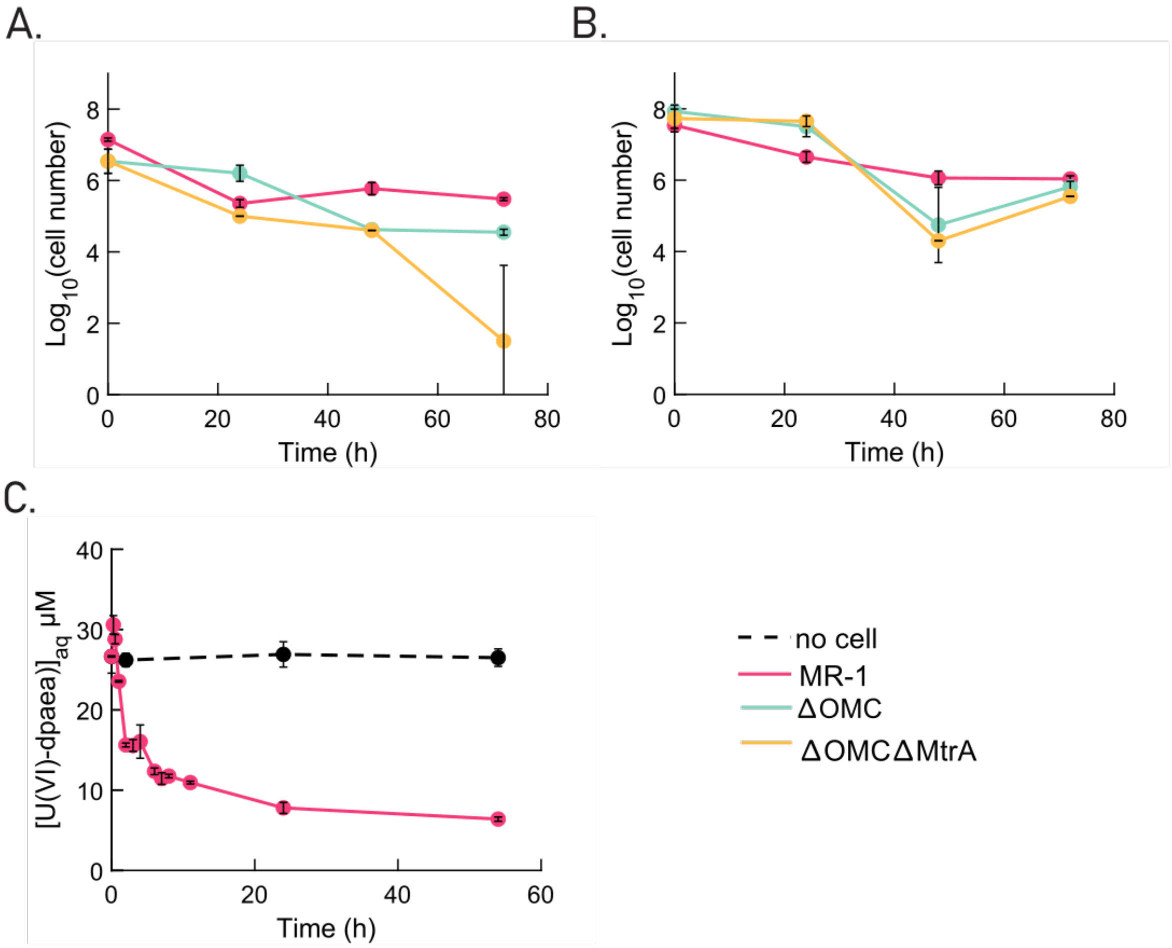

Figure S3: Cell viability of MR-1 (pink dots), ΔOMC (blue dots) and ΔOMCΔMtrA (yellow dots) over the experimental time in incubations with A. solid phase U(VI)-dpaea and B. aqueous U(VI)-dpaea. C. Concentration of U(VI)-dpaea in solution for a no-cell control (black) and MR-1 (pink).

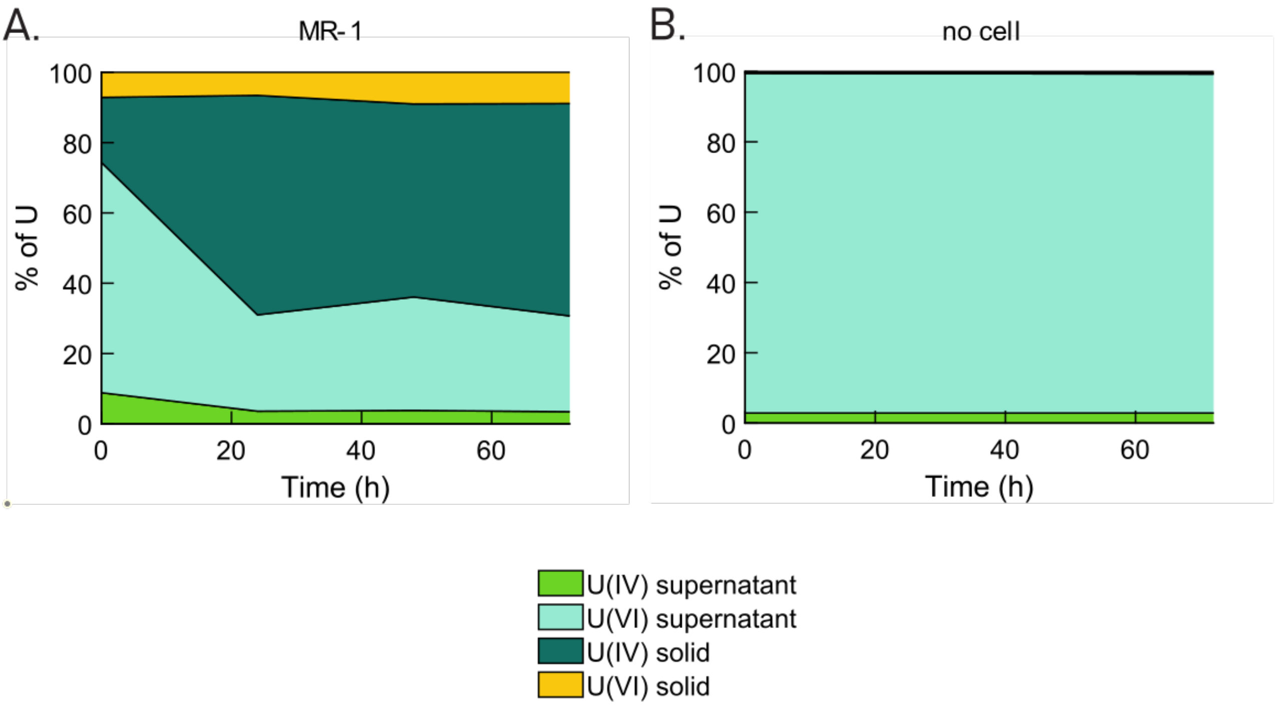

Figure S4: U oxidation state in the solid phase (cell pellet) and in the aqueous phase (supernatant) of (A) incubations with *S. oneidensis* MR-1 (B) and no-cell controls, incubated with 20μM aqueous U(VI)-dpaea, at pH 7.3, OD_{600} =1. The U(VI) and U(IV) fractions were obtained by ion exchange chromatography. The ion exchange chromatography separation cannot directly identify U(V), because the samples are acidified prior to loading onto the column. Acid treatment is known to disproportionate uranyl(V) and to produce equal proportions of U(VI) and U(IV). Therefore, here the equal proportions observed for U(VI) and U(IV) in the supernatant are a proxy for U(V) (result demonstrated by U M_4-edge HR-XANES).

In the controls with no cell, U(VI)-dpaea remains in the aqueous phase over the experimental time With MR-1, we observed that some U is found in the cell pellet, up to 70% of the total U after 72h, however it is mostly solid U(IV) in dark green (about 50%). The solid U(VI) in yellow associated with cells likely represents U(V) (20%). Indeed, upon acidification of the samples before the IEC tests U(V) disproportionates. The remaining U in the aqueous phase

appears as a mixture of aqueous U(VI) in light blue and little U(IV) in green. We interpret this as a mixture of 20% unreduced soluble U(VI)-dpaea and 10% reduced aqueous U(V). Hence it is reduction which is responsible for the decrease of U observed Figure 1.B..

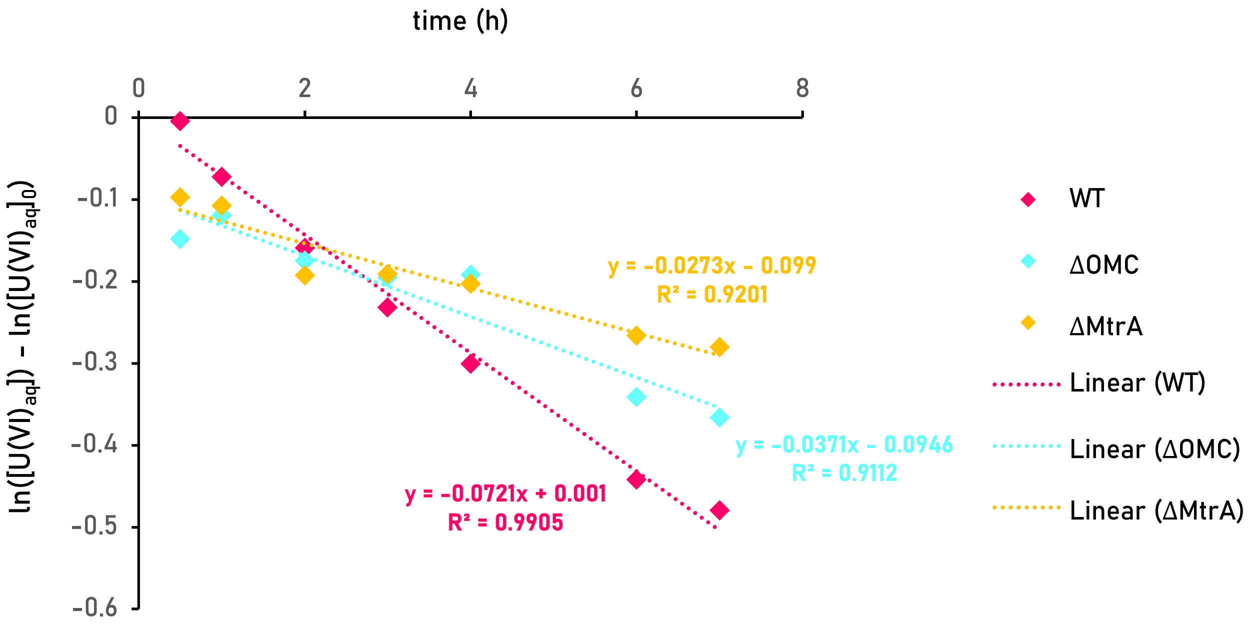

Figure S5: Initial rate of reaction for aqueous U(VI)-dpaea incubated with WT *S. oneidensis* MR-1 (pink), ΔOMC (blue) and ΔOMCΔMtrA (ΔMtrA, for short, in yellow). The data are only considered for the first 7h of the experiment. The corresponding regression equation and the R^2 coefficient are displayed using the same color code.

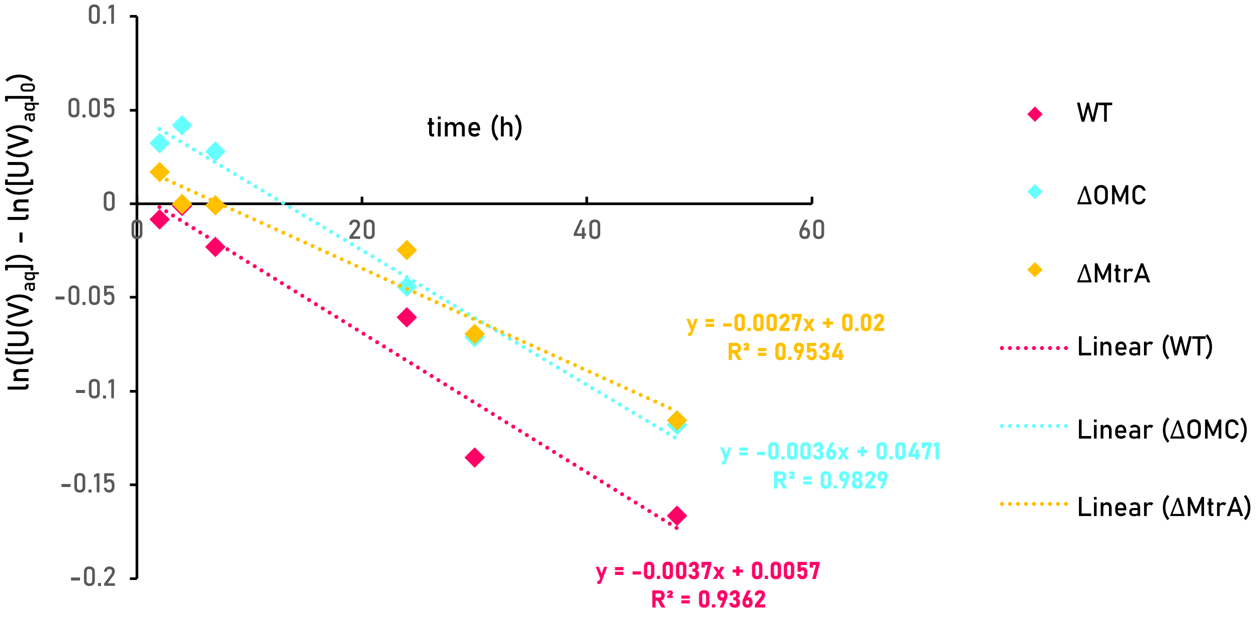

Figure S6: Initial rate of reaction of aqueous U(V)-dpaea incubated with WT *S. oneidensis* MR-1 (pink), ΔOMC (blue) and ΔOMCΔMtrA (ΔMtrA, for short, in yellow). The data are only considered for the first 48h of the experiment. The corresponding regression equation and the R^2 coefficient are displayed using the same color code.

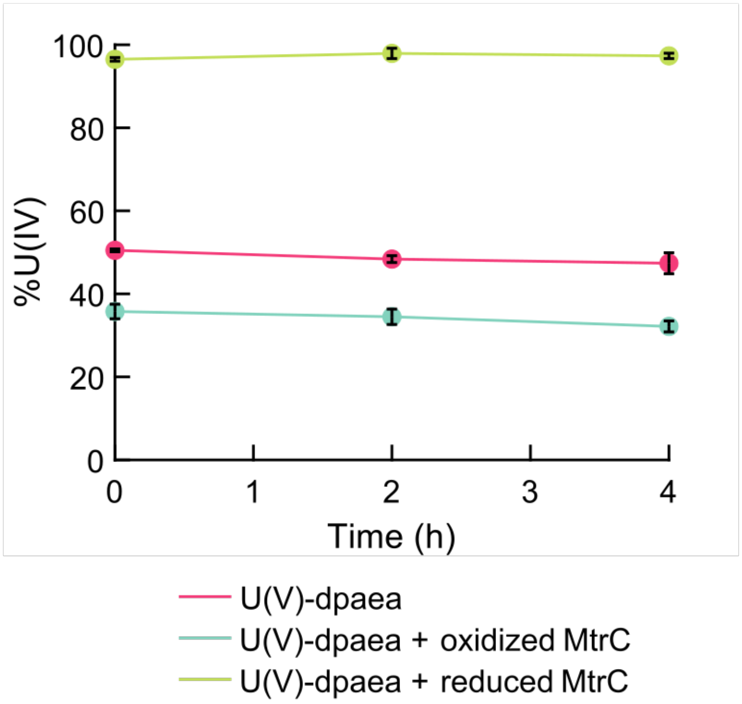

Figure S7: Percentage of U(IV) obtained by ion exchange chromatography of the reaction between U(V)-dpaea and either oxidized (blue) or reduced (green) MtrC after 20s, 2h and 4h of reaction. U(V)-dpaea (pink) was used as a control to ensure that no spontaneous disproportionation occurred during the experimental time. The ion exchange chromatography separation cannot directly identify U(V), because the samples are acidified prior to loading onto the column. Acid treatment is known to disproportionate uranyl(V) to produce equal proportions of U(V) and U(IV). Therefore, here, the equal proportions observed for U(VI) and U(IV) in the supernatant are a proxy for U(V) (result demonstrated by U M_4-edge HR-XANES).

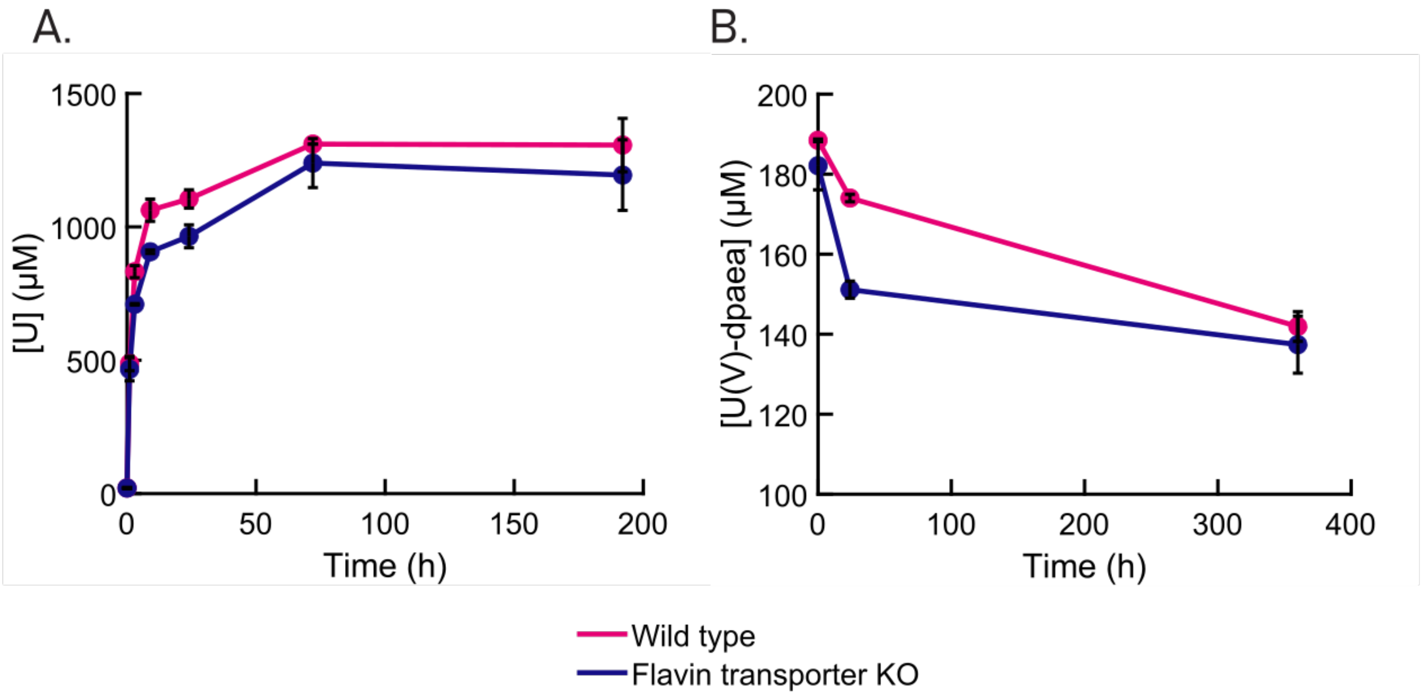

Figure S8: Incubation of the WT MR-1 strain (pink) and the Δ*bfe* strain lacking the flavin transporter system (dark blue) with A. solid U(VI)-dpaea, B. aqueous U(V)-dpaea. This later strain cannot export flavins extracellularly. Both strains were incubated and sampled as described in the paragraph "reduction of U(V)" in the experimental methods section of the main text.

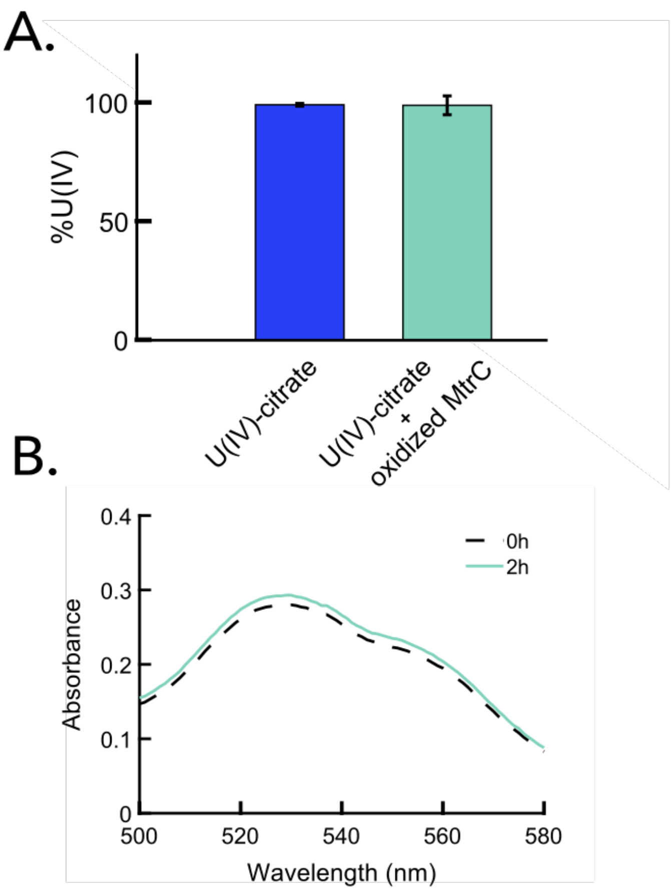

Figure S9: **A.** Percentage of U(IV) obtained by ion exchange chromatography of the reaction between 100μM U(IV)-citrate and 100μM oxidized MtrC (light blue) after 2 min of reaction. U(IV)-citrate in buffer A (dark blue) was used as a control for the U oxidation state. The pH value of the enzymatic reaction between U(IV)-citrate and oxidized MtrC was set to 7.5. **B.** UV-vis spectra of the hemes of MtrC before (dotted black) and after (light blue) reaction (2 hours) with U(IV) citrate, recorded in anaerobic quartz cuvettes.

To probe the oxidation of U(IV) by oxidized MtrC, we selected to consider a soluble form of U(IV) (U(IV)-citrate) rather than insoluble U(IV), formed from the reduction of U(V)-dpaea. In the reaction between oxidized MtrC and U(IV)-citrate, no change was observed in U oxidation state (Figure 4.A., Table S5), nor heme redox status (Figure 4.B.). Hence, we concluded that no electron transfer occurred from U(IV)-citrate to oxidized MtrC.

Primer name	Nucleotide sequence (5'-3')[a]	Use
mtrF_5'I	CGAGTTAGTTTATTGGATGGACTGCAAACTTATTCATAATTCTATCC	Deletion construct
mtrF_5'O	CCGGAATTCTCAACGCCTGATTGGTATAGTAC	Deletion construct
mtrF_3'O(DD)[b]	GGAAATAGAATTCCCCAAGG	Deletion construct
mtrF_3'I	GTCCATCCAATAAACTAACTCG	Deletion construct
mtrF_FO	TAACCATACATCTGTCGGAC	Verification deletion
mtrF_RO(DD)	GGCTTCCCAATTTGTCCCAA	Verification deletion

[a]*Eco*RI sites used for cloning are underlined.

[b] *Eco*RI site in primer mtrF_3'O(DD) and used for cloning occurs in the amplified genomic sequence

Table S1: Primers used for PCR and sequencing for the Δ*mtrC/omcA/mtrF* construct.

Primer name	Nucleotide sequence (5'-3')[a]	Use
mtrA_5'I	GTCTCCTTAGCGCTGTAATAGTAGGCAGTTCTTCATAATAGGC	Deletion construct
mtrA_5'O	CGGGGTACCATCTGAATCGAGAGACGAAAC	Deletion construct
mtrA_3'O	CGGGGTACCGCTGTCCATCAAGAGTGGC	Deletion construct
mtrA_3'I	CTATTACAGCGCTAAGGAGAC	Deletion construct
mtrA_FO	GTTAGCCTTACAGGTGGG	Verification deletion
mtrA_RO	CCACAGGGATTCACCTTG	Verification deletion

[a]*Kpn*I sites used for cloning are underlined

Table S2: Primers used for PCR and sequencing for the Δ*mtrC/omcA/mtrF/mtrA* construct.

	k (s^{-1})	R^2
aqueous U(VI)-dpaea		
**WT**	0.0721	0.99
**ΔOMC**	0.0371	0.91
**ΔOMC/ΔMtrA**	0.0273	0.92
aqueous U(V)-dpaea		
**WT**	0.0037	0.94
**ΔOMC**	0.0036	0.98
**ΔOMC/ΔMtrA**	0.0026	0.94

Table S3: First-order kinetic constants for incubations of aqueous U(VI)-dpaea or U(V)-dpaea with WT, ΔOMC and ΔMtrA *S. oneidensis* MR-1 strains.

Substrate	Reaction mixture	%U(IV)	Standard deviation	[U] (μM)	[MtrC] (μM)	[U]/[MtrC]
U(V)-dpaea	no protein control	47.62	1.72	117.70	/	/
	Oxidized MtrC	33.24	3.19	116.78	166.26	0.70
	Reduced MtrC	98.02	0.81	120.51	155.70	0.77
	dialysis control	49.59	2.55	119.52	/	/
U(IV)-citrate	no protein control	99.02	0.56	113.39	/	/
	Oxidized MtrC	98.78	3.97	113.71	102.20	1.15
solid U(VI)-dpaea	no protein control	4.32	4.25	137.78	/	/
	Reduced MtrC	57.6	2.27	153.87	166.06	0.7
soluble U(VI)-dpaea	no protein control	18.27	1.88	22.07	/	/
	Reduced MtrC	76.21	0.46	23.1	115.24	0.2

Table S4: Summary of the experimental results describing the reactions of U(V)-dpaea with either oxidized or reduced MtrC after 2 min, and also with the dialysis control (no protein control) obtained by dialyzing the amount of sodium dithionite used to reduce MtrC. Additionally, the reaction of U(IV)-citrate with oxidized MtrC is reported. We also included the results from the reaction between reduced MtrC and solid or soluble U(VI)-dpaea. The table also reports the concentration of U, the concentration of MtrC, and their ratio.

Reaction mixture	Time	%U(IV)	Standard deviation	[U] (uM)	[MtrC] (uM)	[U]/[MtrC]
no protein control + U(V)-dpaea	20s	50.48	0.33	129.33	/	/
	2h	48.37	0.82			
	4h	47.37	2.53			
Oxidized MtrC + U(V)-dpaea	20s	35.75	1.76	126.01	175.84	0.72
	2h	34.48	1.85			
	4h	32.15	1.33			
Reduced MtrC + U(V)-dpaea	20s	96.49	0.43	124.54	161.46	0.77
	2h	97.92	1.24			
	4h	97.32	0.64			

Table S5: Summary of the experimental results corresponding to Figure S7 describing the reactions of U(V)-dpaea with either oxidized or reduced MtrC over 4h. The table also reports the concentration of U(V)-dpaea, the concentration of MtrC, and their ratio.

substrate	Apparent [U(IV)]	Apparent [U(VI)]	Deconvoluted [U(IV)]	Deconvoluted [U(V)]
solid U(VI)-dpaea	88.6 µM<br>57.6%	65.3 µM<br>42.4%	23.39 µM<br>15.2%	130.5 µM<br>84.8%
aqueous U(VI)-dpaea	17.6 µM<br>76.2%	5.5 µM<br>23.8%	13 µM<br>52.4%	10.1 µM<br>47.6%
aqueous U(V)-dpaea	118 µM<br>98%	2.51 µM<br>2%	115.49 µM<br>96%	5.02 µM<br>4%

Table S6: U(VI) and U(IV) concentrations and percentage obtained by ion exchange chromatography (two left columns) and proposed derived U(IV) and U(V) concentrations and percentage (two right columns) in the reduction of solid and aqueous U(VI)-dpaea, and aqueous U(V)-dpaea by purified and reduced MtrC after 2 minutes. The ion exchange chromatography separation cannot directly identify U(V), because the samples are acidified prior to loading onto the column. Acid treatment is known to disproportionate uranyl(V) and to produce equal proportions of U(VI) and U(IV).

substrate	time (s)	Deconvoluted U(IV) (µM)	%U(IV)	rate (%.s^{-1})	Rate (µM.s^{-1})
solid U(VI)-dpaea	120	23.39	15.2	0.126	0.19
aqueous U(VI)-dpaea	120	13	52.4	0.437	0.11
aqueous U(V)-dpaea	120	115.49	96	0.8	0.80

Table S7: Apparent formation rates of U(IV) species upon reduction of solid U(VI)-dpaea, aqueous U(VI)-dpaea, or aqueous U(V)-dpaea by purified and reduced MtrC after 2 min. We used the normalized concentration of U(IV), in percent, to calculate the rate in percent per second.

Primer name	Nucleotide sequence (5'-3')	Use
bfe_5'I	CATGGAGAAAATAGTGCCTTG	Deletion construct
bfe_5'O[a]	CGC<u>GGATCC</u>TGGATTAACCTACGATGGCC	Deletion construct
bfe_3'O[b]	CGG<u>GGTACC</u>CTAAGCTCACGGTGGAAGTG	Deletion construct
bfe_3'I	ACAAGGCACTATTTTCTCCATGTAGCATTTAGGTGACACTTTTAA	Deletion construct
bfe_FO	AGCTGCCATAATTGAGTATCC	Verification of deletion
bfe_RO	CTATCCACAGTATGAATGTAC	Verification of deletion

[a] BamHI site is underlined

[b] KpnI site is underlined

Table S8: Primers used for PCR and sequencing for the Δ*bfe* construct.

	Time (h)	Riboflavins (nM)	Standard deviation
Wild Type	0	0.00	0.00
	24	19.45	0.59
	72	39.43	2.25
Δ*bfe*	0	0.00	0.00
	24	9.71	1.76
	72	29.24	3.58

Table S9: Riboflavins profile over 72h measured by HPLC for both the WT and Δ*bfe* when incubated with 4mM U(VI)-dpaea.

References:

(1) Meibom, K. L.; Cabello, E. M.; Bernier-Latmani, R. The Small RNA RyhB Is a Regulator of Cytochrome Expression in Shewanella Oneidensis. *Front. Microbiol.* **2018**, *9*. https://doi.org/10.3389/fmicb.2018.00268.

(2) Molinas, M.; Faizova, R.; Brown, A.; Galanzew, J.; Schacherl, B.; Bartova, B.; Meibom, K. L.; Vitova, T.; Mazzanti, M.; Bernier-Latmani, R. Biological Reduction of a U(V)–Organic Ligand Complex. *Environ. Sci. Technol.* **2021**, *55* (8), 4753–4761. https://doi.org/10.1021/acs.est.0c06633.

(3) Faizova, R.; Scopelliti, R.; Chauvin, A.-S.; Mazzanti, M. Synthesis and Characterization of a Water Stable Uranyl(V) Complex. *J. Am. Chem. Soc.* **2018**, *140* (42), 13554–13557. https://doi.org/10.1021/jacs.8b07885.

(4) Shi, L.; Chen, B.; Wang, Z.; Elias, D. A.; Mayer, M. U.; Gorby, Y. A.; Ni, S.; Lower, B. H.; Kennedy, D. W.; Wunschel, D. S.; Mottaz, H. M.; Marshall, M. J.; Hill, E. A.; Beliaev, A. S.; Zachara, J. M.; Fredrickson, J. K.; Squier, T. C. Isolation of a High-Affinity Functional Protein Complex between OmcA and MtrC: Two Outer Membrane Decaheme c-Type Cytochromes of Shewanella Oneidensis MR-1. *J. Bacteriol.* **2006**, *188* (13), 4705–4714. https://doi.org/10.1128/JB.01966-05.

(5) Kotloski, N. J.; Gralnick, J. A. Flavin Electron Shuttles Dominate Extracellular Electron Transfer by Shewanella Oneidensis. *mBio* **2013**, *4* (1), e00553-12. https://doi.org/10.1128/mBio.00553-12.

(6) Ross, D. E.; Brantley, S. L.; Tien, M. Kinetic Characterization of OmcA and MtrC, Terminal Reductases Involved in Respiratory Electron Transfer for Dissimilatory Iron Reduction in Shewanella Oneidensis MR-1. *Appl. Environ. Microbiol.* **2009**, *75* (16), 5218–5226. https://doi.org/10.1128/AEM.00544-09.
